# Sustainable diets and cancer: a systematic review and meta-analysis

**DOI:** 10.1016/j.eclinm.2025.103215

**Published:** 2025-04-29

**Authors:** Marina Kasper, Mirna al Masri, Tilman Kühn, Sabine Rohrmann, Katharina Wirnitzer, Michael Leitzmann, Carmen Jochem

**Affiliations:** a Department of Epidemiology and Preventive Medicine, University of Regensburg, Regensburg, Germany; b Department of Planetary & Public Health, University of Bayreuth, Bayreuth, Germany; c Center for Public Health, Medical University of Vienna, Vienna, Austria; d Department of Nutritional Sciences, University of Vienna, Vienna, Austria; e Heidelberg Institute of Global Health (HIGH), Medical Faculty and University Hospital, Heidelberg University, Heidelberg, Germany; f Institute of Global Food Security (IGFS), Queen's University Belfast, Belfast, UK; g Division of Chronic Disease Epidemiology, Epidemiology, Biostatistics and Prevention Institute, University of Zurich, Zurich, Switzerland; h Department of Pediatric Oncology and Hematology, Otto-Heubner Centre for Paediatric and Adolescent Medicine (OHC), Charité – Universitätsmedizin Berlin, Augustenburger Platz 1, Berlin, 13353, Germany; i Charité Competence Center for Traditional and Integrative Medicine (CCCTIM), Charité – Universitätsmedizin Berlin, Berlin, Germany; j Department of Sport Science, Leopold-Franzens University of Innsbruck, Fürstenweg 185, Innsbruck, 6020, Austria; k Department of Secondary Education, University College of Teacher Education Tyrol, Pastorstraße 7, Innsbruck, 6010, Austria

**Keywords:** Sustainable diets, Cancer prevention, Cancer incidence/mortality, Dietary sustainability indices, Planetary health

## Abstract

**Background:**

Sustainable diets are increasingly recommended as a strategy to reduce non-communicable diseases and promote planetary health. Current unhealthy dietary patterns are thought to contribute to the global cancer burden while food systems continue to exacerbate environmental challenges. Investigating the impact of sustainable diets on cancer is therefore critical.

**Methods:**

This systematic review and meta-analysis included observational studies of healthy adults at baseline, reporting cancer incidence or cancer mortality during follow-up. Eligible studies were identified through a comprehensive search of multiple databases, including PubMed, ISI Web of Science, EMBASE, and the Cochrane Library from inception to February 28, 2025. Sustainable diets were assessed using various metrics, and effect measures were pooled to compare adherence to sustainable dietary patterns. Summary effect estimates for cancer incidence and mortality were calculated using random-effects models. Subgroup analyses were conducted for sex, geographic regions, study design, sustainability metrics, dietary assessment indices on sustainability, cancer types, and dietary energy intake adjustment. E-values were used to assess the robustness of associations against potential unmeasured confounding. The study was pre-registered in PROSPERO (ID CRD42024545102).

**Findings:**

We pooled 19 effect estimates from 17 studies, identified through the literature search. These studies encompassed over 2·2 million participants, with studies spanning from 1983 to 2022. Adherence to sustainable diets revealed a significant reduction in cancer incidence (RE = 0·93 [95% CI 0·88–0·98], I^2^ = 84·67%) and cancer mortality (HR = 0·88; 95% CI 0·85–0·92, I^2^ = 21·25%). Subgroup analyses indicated that the overall effect was modified by study region and design, sustainability metrics and dietary assessment indices. High heterogeneity, risk of bias in some studies, and e-values indicating potential residual confounding resulted in an overall low level of evidence as evaluated using GRADE.

**Interpretation:**

These findings provide pooled evidence linking sustainable diets to reduced cancer incidence and mortality, highlighting their potential for cancer prevention and their dual health and environmental benefits. This analysis also revealed notable differences in sustainability metrics, emphasizing the need for standardized approaches.

**Funding:**

This research received no external funding.

**Research in context**

**Evidence before this study**

Before undertaking this meta-analysis and systematic review, the authors systematically reviewed existing literature on the relationship between dietary patterns, particularly plant-based and sustainable diets, and their impact on non-communicable diseases (NCDs), including cancer, cardiovascular disease, diabetes, and obesity. The sources searched included PubMed, ISI Web of Science, EMBASE, and Cochrane libraries databases, covering studies published up to February 28, 2025. The search terms used keywords such as “cancer”, “planetary health”, “sustainable diet”, and “environmental footprint”. Studies were included if they met the following criteria: (1) observational design (prospective cohort, case-control, or cross-sectional); (2) examined the relationship between adherence to sustainable diets and cancer incidence or mortality; (3) reported effect estimates (odds ratio (OR), relative risk (RR), or hazard ratio (HR)) with 95% confidence intervals (CI); and (4) provided data from adult participants free of cancer at the time of study enrollment.

**Added value of this study**

This review examines the relationship between sustainable diets and cancer outcomes while also reinforcing findings from previous research on other non-communicable diseases (NCDs). By examining the effects of sustainable diets on cancer incidence and mortality, alongside evidence from previous meta-analyses on cardiovascular disease, obesity, and diabetes, this review presents a holistic framework linking health benefits with environmental sustainability.

**Implications of all the available evidence**

The combined evidence suggests that sustainable diets offer substantial benefits for individual health, including lower risks of cancer and other NCDs, while also contributing to environmental sustainability. These findings underscore the need for public health policies promoting sustainable diets as a strategy to reduce the global burden of cancer and other NCDs.

## Introduction

From a planetary health perspective—which refers to “the health of human civilization and the state of the natural systems on which it depends”^1^—nutrition and cancer are intricately linked at various levels.^2^^,^^3^ On one hand, the food system is a major contributor to the triple planetary crisis, encompassing climate change, biodiversity loss, and pollution. Globally, up to 40% of land is utilized for agriculture, about 70% of fresh water is used for food production, and approximately 30% of worldwide greenhouse gas emissions stem from food processing.^4^, ^5^, ^6^ On the other hand, unhealthy dietary choices are associated with the development of various types of cancer, including aerodigestive, esophageal, stomach, pancreatic, colorectal, endometrial, and breast cancers.^7^^,^^8^ The Global Burden of Disease (GBD) Study characterized unhealthy food components by excess energy intake, high levels of salt, sugar, and saturated fats, high red meat consumption and pronounced proportions of ultra-processed foods, as well as insufficient intake of dietary fiber and healthy food items like fruits, vegetables, legumes, whole grains.^7^^,^^8^ Taking into consideration (1) current diets often characterized by unhealthy and unsustainable choices^4^^,^^7^^,^^8^; (2) a projected 77% increase in cancer incidence by 2050 due to demographic changes^9^; and (3) detrimental effects of the food system on planetary health,^4^, ^5^, ^6^^,^^10^, ^11^, ^12^ it is increasingly relevant to investigate the role of nutrition as a major modifiable risk factor in cancer prevention from a planetary health perspective.^1^^,^^2^^,^^7^^,^^13^^,^^14^

To achieve a healthy diet for an estimated global population of 10 billion by 2050, the EAT-Lancet Commission offers a holistic scientific framework aimed at mitigating the environmental instability caused by food systems.^1^^,^^4^^,^^6^ Similarly, the Food and Agriculture Organization of the United Nations (FAO) defines sustainable diets as those that are environmentally beneficial, culturally adaptive, socially and economically equitable, nutritionally adequate, and protective of both human and planetary resources, integrating several United Nations Sustainable Development Goals (SDGs).^15^^,^^16^

In recent years, convincing evidence has emerged on sustainable nutrition in the context of non-communicable disease (NCD) prevention. For instance, a meta-analysis and systematic review reported a relative risk (RR) of 0·69 [95% CI 0·62–0·76] for overweight and 0·61 [95% CI 0·47–0·78] for obesity associated with adherence to sustainable diets.^17^

Recent observational studies on sustainable nutrition and cancer now enable a systematic review with updated evidence and quantitative analyses to follow up the work by Karavasiloglou et al.^18^ Recently published systematic reviews and meta-analyses have focused on the association between adherence to the recommended EAT-Lancet Diet or organic food consumption and health outcomes including cancer.^19^, ^20^, ^21^ In contrast, the aim of the present work was to go beyond these specific dietary behaviors by investigating the associations of broader aspects of sustainable diets in relation to and cancer incidence and mortality. Additionally, sensitivity analyses were performed to identify potential moderators, such as varying sustainability assessment metrics and dietary sustainability scores. To the best of the authors' knowledge, this is the first systematic review and meta-analysis to address this subject, drawing on the latest available research. Based on the relevant literature available and the previous work on this research question, it was hypothesized that following a recommended sustainable diet is associated with reduced cancer outcomes due to health-promoting properties of the recommended food constituents.

## Methods

### Search strategy and selection criteria

The present systematic review and meta-analysis was conducted with methodological orientation based on the updated 2020 guidelines for reporting systematic reviews (PRISMA; Supplemental Table S1).^22^ The study protocol was registered a priori at PROSPERO under the ID CRD42024545102 (https://www.crd.york.ac.uk/prospero/display_record.php?RecordID=545102). To be included, studies needed to meet the following criteria: (1) Observational study with a prospective cohort, case-control, case-cohort, or cross-sectional design; (2) examined the relationship between adherence to a sustainable diet, consumption of organic food, and cancer risk, cancer recurrence, and cancer-specific mortality; (3) reported effect estimates in the form of odds ratio (OR), relative risk (RR), or hazard ratio (HR), along with corresponding 95% confidence intervals (CI); (4) included data from healthy adult participants at the time of study enrollment; (5) reported cancer outcomes that were either physician-confirmed, self-reported or based on cancer or mortality registries; and (6) published in English. Moreover, the following eligibility criteria were defined a priori for this review: (i) Diets were defined as sustainable if they were assessed using existing sustainability indices or self-computed sustainability scores; (ii) organic food consumption and diets that provided information on ecological footprints were considered indicative of a sustainable diet^23^, ^24^, ^25^, ^26^; (iii) conventional, predominantly plant-based diets, such as the Mediterranean diet, were excluded unless their sustainability was explicitly measured using a recognized index or measure; and (iv) studies involving animal or cell models were also excluded.

A systematic literature search was conducted using the scientific databases PubMed, EMBASE, ISI Web of Science, and Cochrane Library, covering the period from inception through February 28, 2025. The pre-defined search terms are detailed in Supplemental Data S1. Additionally, relevant references from identified studies and manually retrieved documents were included in the review.

### Ethics

This systematic review and meta-analysis does not involve human or animal subjects or other ethical considerations requiring declaration.

### Statistics

Literature research was conducted in duplicate by M.K. and C.J. Results were screened for titles and abstracts, and full texts were assessed for eligibility if deemed relevant. Duplicate records were manually identified and removed using *Microsoft Excel*. Decisions regarding the inclusion of full-text papers were discussed between M.K. and C.J., and any discrepancies were resolved through consensus. Data extraction was performed by M.K., focusing on the following characteristics: author name, publication year, study name and design, geographic region, sample size, participant age and sex distribution, follow-up period, methods of dietary and sustainability assessments, effect estimates (hazard ratios [HR], odds ratios [OR], or relative risks [RR]) with corresponding 95% confidence intervals [CIs], reported outcomes (e.g., cancer-specific incidence or cancer), and covariates adjusted for in the studies. Data extraction was carried out in duplicate by M.M., and any inconsistencies were resolved through discussion with C.J.

Effect estimates and their 95% CIs were extracted based on comparisons between the highest and lowest levels of adherence to sustainable diets. For three studies, inverse estimates were re-calculated.^27^, ^28^, ^29^ One effect estimate per study was included in the primary analysis, with sustainable diets as the defined exposure. To address studies reporting multiple sustainability metrics^27^^,^^28^ and for selection of only one study representing a cohort mentioned in multiple publications we used a pre-defined hierarchy based on the most comprehensive methodology of sustainability metrics^4^^,^^30^: 1) Measurements or indices based on dietary behavior (e.g., Eat Lancet Index, Planetary Health Diet Index) that address both human and planetary health; 2) Greenhouse gas (GHG) emissions, reflecting global environmental impacts; 3) land use (LU), indicating regional environmental burdens; 4) food biodiversity, measured as dietary species richness (DSR); and 5) organic food consumption, reflecting agricultural practices. For analysis, the most comprehensively adjusted effect estimates were extracted. Overall estimates were calculated separately for cancer incidence and cancer mortality using the natural logarithm of OR, RR, or HR, with variances derived from the squared standard errors. Standard errors were calculated by subtracting the natural logarithm of the effect estimate (logOR, logRR, or logHR) from the upper and lower bounds of the 95% CI. A random effects model was applied due to heterogeneity across the included studies. Heterogeneity was assessed using the I^2^ and Q statistics, with τ^2^ estimated through restricted maximum likelihood methods.^31^^,^^32^

The risk of bias was evaluated using tools for non-randomized follow-up studies of exposure effects (ROBINS-E).^33^ Risk of bias assessments were performed independently by M.K. and M.M., and disagreements were resolved through discussion. To assess confounding, relevant adjustment factors were pre-defined, including sex, age, socioeconomic status, physical activity, body mass index (BMI), energy intake, fiber intake, dietary habits, alcohol consumption, smoking status, family history of cancers or chronic diseases, and, for women, parity, age at first birth, menopausal status, hormonal treatment use (post-menopause or contraception), and age at menarche. The overall risk of bias was visualized using *robvis*.^34^ We used *GRADE* to assess the certainty of evidence across studies.^35^

The risk of publication bias was addressed using funnel plot methods, along with trim-and-fill analysis, Egger's regression, and Begg's rank correlation test.^36^^,^^37^ To assess the impact of individual studies on the overall results, influence diagnostics and leave-one-out analysis were applied.^38^ Sensitivity analyses were performed using e-values to quantify the robustness of observed associations against unmeasured confounding factors.^39^ Cancer incidence and mortality analyses were stratified by geographic region, sex, study design, sustainability assessment method (e.g., sustainable dietary patterns, GHG, LU, food biodiversity, and organic food consumption), cancer type, and dietary energy intake adjustment. Additionally, sensitivity analyses were conducted for dietary indices described in the studies as the applied scores to quantify dietary sustainability distinct from each other (e.g., Planetary Health Diet Index,^40^ EAT-Lancet Diet Index^41^) and for scoring systems of applied indices (continuous, binary, proportional, and ordinal). All statistical analyses were performed using R software (version 4.2.3; R Core Team, Vienna, Austria; www.R-project.org/), employing the packages metafor (version 4.8-0), robumeta (version 2.1), EValue (version 4.1.3), and dplyr (version 1.1.4). p-values < 0·05 were considered statistically significant.^42^, ^43^, ^44^, ^45^

### Role of funding source

For this present study, the authors declare no role of funding.

## Results

### Study selection and study participants

**Fig. 1:**
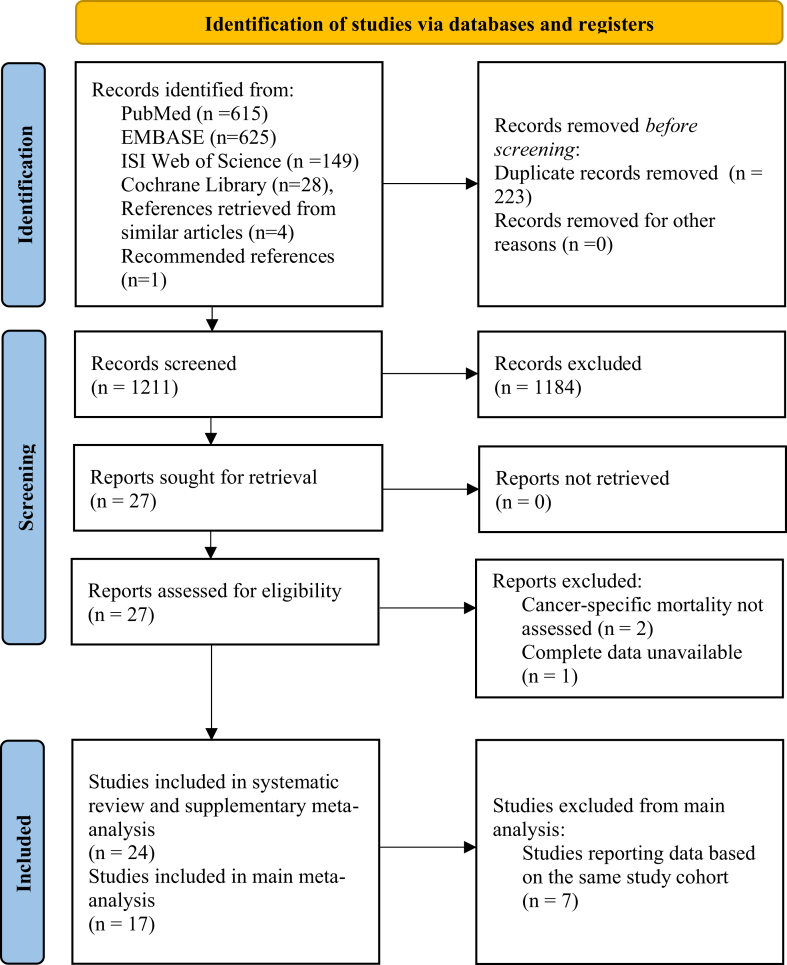
**PRIMSA flowchart**.^22^

We obtained a total 1211 search results after removing duplicates from the electronic databases PubMed EMBASE, ISI Web of Science, and Cochrane Library, as well as manually screening of references and recommendations (Fig. 1). After screening the titles and abstracts of these results, 1184 publications were excluded, leaving 27 studies for full-text review. Two studies were excluded due to missing data on cancer-specific mortality outcomes^46^^,^^47^ and for lacked complete data.^48^ Ultimately, 24 studies met the eligibility criteria and were included in our systematic review and for supplementary meta-analysis, 17 studies were included for main quantitative analysis of this meta-analysis.

Table 1 summarizes the main characteristics of the included studies. 22 studies are prospective cohort studies,^19^^,^^23^^,^^27^, ^28^, ^29^, ^30^^,^^40^^,^^41^^,^^49^, ^50^, ^51^, ^52^, ^53^, ^54^, ^55^, ^56^, ^57^, ^58^, ^59^, ^60^, ^61^, ^62^, ^63^ and two are case-control studies.^64^^,^^65^ 16 studies were conducted in Europe,^27^, ^28^, ^29^^,^^41^^,^^49^, ^50^, ^51^^,^^53^, ^54^, ^55^^,^^57^, ^58^, ^59^^,^^62^^,^^66^^,^^67^ three in Asia,^61^^,^^64^^,^^65^ and five in North America.^40^^,^^52^^,^^56^^,^^60^^,^^63^ Due to variations in standard errors from different sample sizes and differences in measurement methods, exposure categorization, covariate adjustments, stratifications, and confidence interval estimation, studies with the same effect sizes may exhibit differences in the lower bounds of their confidence intervals.^27^^,^^61^ For three studies that reported a value of 1·00 within the boundaries of the 95% confidence interval, it should be noted that the authors considered this value to be included within the effect estimates.^27^^,^^50^^,^^61^ Notably, multiple studies used data from the same study populations: Four studies used data from the NutriNetSanté cohort,^41^^,^^57^^,^^58^^,^^66^ three studies are based on the EPIC cohort,^28^^,^^51^^,^^62^ two studies report data from the Prostate, Lung, Colorectal and Ovarian Cancer (PLCO) Screening Trial,^56^^,^^60^ and Karavasiloglou et al.^53^ and Liu et al.^67^ refer to the UK Biobank cohort. To avoid potential dependence between effect estimates from studies based on the same cohort, we included only one study per cohort for the main analyses. Study inclusion was guided by a pre-defined hierarchy of sustainability metrics, prioritizing methodological comprehensiveness and study quality rather than outcome desirability. Following this strategy, we included the study by Berthy et al.^41^ for the main analysis of cancer incidence effect estimates, as it represents the NutriNet-Santé cohort and employs the Eat Lancet Diet Index to assess dietary sustainability. For the EPIC cohort, we selected the study by Laine et al.^28^ which provided a more precise sustainability metric based on greenhouse gas emissions, compared to the food biodiversity measure used in the study by Hanley-Cook et al.^51^ and in the more recent study by Huybrechts et al.^62^ Regarding the PLCO cohort, where dietary sustainability was assessed via adherence to the EAT Lancet Diet, we included Xiao et al., as it provided data on both cancer incidence and mortality, thus contributing two relevant endpoints.^60^ For the UK Biobank cohort where dietary sustainability behavior was captured using the EAT Lancet Index, we included the study by Karavasiloglou et al.,^53^ as it reported all-cause cancer outcomes.

**Table 1:** Main characteristics of the 24 included studies assessing sustainable diets and cancer incidence/mortality.

Author, year	Geographic region, study population, recruitment period	Analytic sample size	Exposure; assessment of exposure	Outcome (95% CI)	Adjusted confounding factors
Andersen, 2023^a^	Denmark; The Danish Diet, Cancer and Health Cohort; 1993–1997; men and women	41,928	Organic food consumption; organic food consumption score (range, 6–24) for six food groups	Cancer incidence HR_Q4 vs. Q1_ 0·99 (0·91–1·08)	Age, smoking, alcohol, educational level, BMI, and physical activity; stratified by sex
Baudry, 2018	France; NutriNet Santé Cohort; 2009–2016; men and women	68,946	Organic food consumption; organic food consumption score of 16 products (range, 0–32)	Cancer incidence HR_Q4 vs. Q1_ 0·76 (0·64–0·90)	Age, sex, month of inclusion, occupational status, educational level, marital status, monthly household income, physical activity, smoking, alcohol, family history of cancer, BMI, height, energy intake, mPNN-GS, fiber intake, processed and red meat intake, ultra processed food intake, fruit and vegetable consumption, dietary patterns extracted by PCA, (for women) parity, postmenopausal status, use of hormonal treatment, and oral contraception
Berthy, 2022^a^	France; NutriNet Santé; 2009–2021; men and women	62,382	Sustainable dietary pattern; EAT-Lancet diet index ELD-I (continuous score)	Cancer incidence HR_Q5 vs. Q1_ 0·98 (0·86–1·12)	Age, sex, energy intake, education level, occupational status, monthly household income, marital status, number of completed 24-h-records, physical activity, smoking, alcohol, height, BMI, family history of cancer, and family history of chronic diseases
Bradbury, 2014^a^	United Kingdom; The Million Women Study; 1996–2001; women	623,080	Organic food consumption; questionnaire on frequency (never, sometimes, usually/always)	Cancer incidence RR_usually/always vs. never_ 1·03 (1·00–1·06)	BMI, height, smoking, alcohol, physical activity, parity, fiber intake, frequency and type of meat consumption, stratified by age, geographical region, and socioeconomic status
Bui, 2024^a^	United States of America; Nurses' Health Study I + II, Health Professions Follow-up Study; 1996–2019; men and women	206,404	Sustainable dietary pattern; Planetary Health Diet Index (PHDI) (range, 0–140)	Cancer mortality HR_Q5 vs. Q1_ 0·90 (0·85–0·95)	Race, marriage status, living status, socioeconomic status, menopausal status (for women only), multivitamin use, aspirin use, energy intake, BMI, smoking, physical activity, hypertension, hypercholesterinemia, family history of myocardial infarction, family history of diabetes, family history of cancer; stratified for age, and follow-up cycle
Gonzales, 2020^a^	Spain; EPIC-Spain; 1992–1998; men and women	40,621	Greenhouse gas emissions in kgCO_2_eq/kg food (continuous)	Cancer incidence HR_T1 vs. T3_ 0·93 (0·86–1·01)	Sex; stratified by age and center
Han, 2025	United States of America; National Health and nutrition Examination Survey; 2005–2018; men and women	30,521	Sustainable dietary pattern; Planetary Health Diet Index for the united States (PHDI-US) (range 0–150)	Cancer mortality HR_Q4 vs. Q1_ 0·68 (0·52–0·89)	Age, sex, NHANES cycle, ethnicity, BMI, energy intake, alcohol, smoking, physical activity, diabetes mellitus, hypertension
Hanley-Cook, 2021	Europe; EPIC; 1992–2014; men and women	451,390	Food biodiversity; Dietary Species Richness (DSR) (continuous)	Cancer mortality HR_Q5 vs. Q1_ 0·75 (0·69–0·82)	Smoking, education, socioeconomic status, marital status, physical activity, alcohol, energy intake, 18-point relative Mediterranean diet score, consumption of red and processed meat, and fiber intake; stratified by sex, age, and center
Huybrechts, 2024	Europe; EPIC; 1992–2002; men and women	521,323	Food biodiversity; Dietary Species Richness (DSR) (continuous)	Gastrointestinal cancer incidence HR_Q5 vs. Q1_ 0·78 (0·69–0·88)	Sex, alcohol, physical activity, marital status, smoking, education, height, BMI, energy intake, calcium intake, fiber intake, 18-point relative Mediterranean diet score consumption of red and processed meat; stratified by sex, age, and center
Karavasiloglou, 2023^a^	United Kingdom; UK Biobank Study; 2006–2010; men and women	473,836	Sustainable dietary pattern; EAT-Lancet reference diet score (range 0–11)	Cancer incidence HR_T3 vs. T1_ 0·91 (0·87–0·95)	Age, sex, region, smoking, alcohol, BMI, physical activity, education, and deprivation
Laine, 2021^a^	Europe; EPIC 1991–2002; men and women	443,991	1) Greenhouse gas emissions in kg CO_2_eq/kg food 2) land use in m^2^/kg food	Cancer incidence 1) GHG HR_Q1 vs. Q4_ 0·90 (0·88–0·92) 2) LU HR_Q1 vs. Q4_ 0·88 (0·87–0·92) Cancer mortality 1) GHG HR_Q1 vs. Q4_ 0·86 (0·81–0·91) 2) LU HR_Q1 vs. Q4_ 0·83 (0·79–0·86)	Age, marital status, education, physical activity, smoking, and BMI
Liu, 2024	United Kingdom; UK Biobank Study; 2006–2010; men and women	175,214	Sustainable dietary pattern; EAT-Lancet Diet Score (range, 0–14)	Lung cancer incidence EAT Lancet Diet Score HR_Q5 vs. Q1_ 0·64 (0·51–0·80) Lung cancer mortality EAT Lancet Diet Score HR_Q5 vs. Q1_ 0·65 (0·48–0·88)	Age, sex, ethnicity, SES, energy intake, smoking, alcohol, physical activity, BMI, polygenetic risk score, ancestry, genotype
Mangone, 2023^a^	Italy; EPIC Italy Cohort; men and women	47,749	1) Sustainable dietary pattern; EAT-Lancet distance index (EatDI) (14-dimensional space) 2) Greenhouse gas emissions in kg CO_2_eq/kg food 3) land use in m^2^/kg food	Cancer incidence 1) EatDI HR_Q1 vs. Q4_ 0·86 (0·77–0·93) 2) GHG HR_Q1 vs. Q4_ 0·93 (0·83–1·00) 3) LU HR_Q1 vs. Q4_ 0·93 (0·83–1·00)	Age, BMI, physical activity, educational level, and smoking; stratified by sex
Mohammadi, 2024^a^	Iran; Case-control study in the Cancer Department of Imam Khomeini Hospital Tehran; men and women	213	Sustainable dietary pattern; Planetary Health Diet Index total score (PHDI) (range, 0–150)	Colorectal cancer incidence OR_T3 vs. T1_ 0·41 (0·18–0·91)	Income, smoking, family history of cancer, intake of ibuprofen, intake of aspirin, intake of acetaminophen
Pitt, 2024^a^	Sweden; The Cohort of Swedish Men, The Swedish Mammography Cohort; 1998–2019; men and women	68,175	Sustainable dietary pattern; EAT-Lancet Diet Adherence Index (EAI) (range, 0–14)	Cancer mortality HR_Q4 vs. Q1_ 0·92 (0·85–0·99)	Age, education, living status, smoking, BMI, walking/cycling, exercise frequency, supplement use, hypertension, hypercholesterinemia, energy intake, and alcohol use
Quartiroli, 2024^a^	Italy; ORDET cohort; 1987–1992; women	9144	Sustainable dietary pattern; EAT-Lancet Score (range, 0–12)	Breast cancer incidence HR_T3 vs. T1_ 1·10 (0·88–1·39)	Age, energy intake, smoking, education, BMI, age at menarche, menopausal status, parity, age at first birth
Rebouillat, 2021	France; NutriNet Santé; 2009–2014 women	13,149	Organic food consumption; dietary pesticide exposure (categorized in pesticide components with non-negative matrix formation (NMF))	Breast cancer incidence HR_Q5 vs. Q1_ 0·92 (0·85–0·99)	Smoking, educational level, physical activity, alcohol, energy intake, BMI, height, family history of cancer, pro-vegetarian score, menopausal treatment, and parity
Ren, 2024	United States of America; PLCO; 1993–2001; men and women	101,755	Sustainable dietary pattern; EAT-Lancet Diet Score (ELD) (range, 0–43)	Head and neck cancer incidence HR_Q4 vs. Q1_ 0·52 (0·34–0·80)	Age, sex, ethnicity, education, family history of cancer, BMI, smoking, alcohol, energy intake
Seconda, 2020	France; NutriNet Santé; 2009–2018 men and women	25,592	Sustainable dietary pattern; Sustainable Diet Index (SDI); seven indicators and four sub-indices (range, 4–20)	Cancer incidence HR_Q4 vs. Q1_ 0·56 (0·41–0·77)	Age, sex, educational status, smoking, household income, occupational status, alcohol, family history of cancer or cardiovascular diseases, physical activity, energy, BMI, (for women) postmenopausal status, use of hormonal treatment, and use of contraception
Shan, 2025^a^	United States of America; The Black Women's Health Study; 1995, women	33,824	Sustainable dietary pattern; Planetary Health Diet Index (PHDI) (range, 0–140)	Cancer mortality HR_Q5 vs. Q1_ 0·91 (0·74–1·12)	Age, energy intake, education, BMI, smoking, alcohol, physical activity; stratified by age, SES, smoking, education, BMI
Stubbendorff, 2022^a^	Sweden; Malmö Diet and Cancer Cohort; 1991–1996 men and women	22,421	Sustainable dietary pattern; EAT-Lancet diet index (range, 0–42)	Cancer mortality HR_Q5 vs. Q1_ 0·76 (0·63–0·92)	Age, sex, dietary assessment version, season, energy intake, BMI, physical activity, smoking, alcohol, and educational status
Xiao, 2023^a^	United States of America; PLCO; 1993–2001; men and women	101,755	Sustainable dietary pattern; Eat-Lancet Diet Score (ELD) (range, 0–14)	Lung cancer incidence HR_Q4 vs. Q1_ 0·73 (0·60–0·89) Lung cancer mortality HR_Q4 vs. Q1_ 0·74 (0·59–0·93)	Sex, age, education, ethnicity, occupation, energy intake, physical activity, BMI, BMI at age 20, weight change, trail arm, smoking, alcohol, use of aspirin, family history of cancer, hypertension, diabetes mellitus, chronic bronchitis, emphysema
Ye, 2023^a^	China; The Singapore Chinese Health Study; 1993–1998; men and women	57,078	Sustainable dietary pattern; Planetary Health Diet Score (PHD-S) (range, 0–140)	Cancer mortality HR_Q5 vs. Q1_ 0·93 (0·86–1·00)	Age, sex, energy intake, dialect group, educational status, BMI, smoking, alcohol, physical activity, sleep duration, hypertension, and diabetes
Zhang, 2023^a^	China; Diet and glioma case-control study at Beijing Tiantan Hospital, Capital Medical University; 2021–2022; men and women	1012	Sustainable dietary pattern; Planetary Health Diet Score (range, 0–150)	Brain cancer incidence OR_T3 vs. T1_ 0·61 (0·34–1·08)	Age, BMI, energy intake, educational status, occupation, household income, high-risk residential areas, alcohol, smoking, physical activity, history of head trauma, allergies, and family history of cancer

***Abbreviations:*** approx., approximately; BMI, Body mass index; CI, confidence interval; GHG, greenhouse gas emissions; HR, hazard ratio; LU, land use; mPNNS-GS, Programme National Nutrition Santé Guideline Score; OR, odds ratio; PCA, principal component analysis; PLCO, Prostate, Lung, Colorectal, and Ovarian Cancer Screening Trial; Q, quartile/quintile; RR, relative risk; SHARP, Sustainable Health and Agriculture Resilience Program; T, tertile.

a Included in the main analysis of the meta-analysis on cancer incidence or mortality.

To enhance transparency and provide additional context, we present the summary effect estimates from all 24 eligible studies retrieved during our literature search in the Supplemental Materials. We also conducted sensitivity analyses to ensure the robustness of the effect estimates when limiting each supplemental model for both cancer incidence and mortality to a single study per cohort. These analyses did not reveal substantial changes in the overall effect estimates (Supplemental Tables S4–S7). In total, data from 3,621,503 participants were included out of which 2,264,134 were considered for outcome estimates within the main analysis of meta-analysis.

### Findings on dietary assessment methods and sustainability metrics

The included studies exhibited heterogeneity in dietary assessment methods and sustainability metrics, relying on a variety of indicators. Dietary assessments were conducted using one or more of the following approaches: food frequency questionnaires administered at baseline or pre-defined intervals, 24-h dietary records, seven-day-records, or data collection on the frequency of organic food consumption. Sustainability indicators reported in these included indices or scores evaluating the sustainability of dietary patterns,^27^^,^^40^^,^^41^^,^^52^, ^53^, ^54^, ^55^, ^56^^,^^58^, ^59^, ^60^, ^61^^,^^63^, ^64^, ^65^^,^^67^ frequency of organic food consumption,^49^^,^^50^^,^^57^^,^^66^ greenhouse gas emissions,^27^, ^28^, ^29^ land use,^27^^,^^28^ and food biodiversity.^51^^,^^62^ To quantify adherence to dietary recommendations published by the EAT Lancet Commission, several indices or scores and terminology of sustainable diets are utilized in current literature. In the studied populations the EAT-Lancet Diet score based on Stubbendorff et al.,^59^ Kesse-Guyot et al.,^68^ and Knuppel et al.^69^ as well as the EAT-Lancet Diet Adherence Index (EAI),^54^ and the EAT-Lancet Diet Distance Index (EatDI)^27^ were applied. Also, the Planetary Health Diet Index (PHD-I) based on Cacau et al.^70^ or Bui et al.^40^ and the Planetary Health Diet Score (PHD-S)^61^ were used for rating adherence to the Planetary Health Diet. A further metric, the Sustainable Diet Index (SDI), was used in one study to capture individual-level sustainability.^58^ Given the variability in the composition of these indices across studies, which considered sustainable diets as the exposure variable, this work includes an assessment of the applied scores of the studies (Table 2). Some of the indices were developed by the authors themselves.

**Table 2:** Overview of sustainable diet assessment indices used in the included studies.

Publication	Score	Primary author	Scoring system	Evaluation (strengths and limitations)	
Berthy et al., 2022^a^	EatLancet Diet Index	Kesse-Guyot et al., 2021	14 food components; score expressed continuous by application of mathematical formula	Greenhouse gas emissions inversely correlating with higher scores^41^^,^^71^	Complexity of interpretation; Higher consumption of emphasized food might balance consumption of restricted food
Bui et al., 2024^a^	Planetary Health Diet Index (PHD-I)	Bui et al., 2024	15 food components; Proportionally scored (0–10 or 0–5) per component, total range 0–140	Flexibility and adaptability^40^	Complexity of Interpretation^40^
Han et al., 2025^a^	Planetary Health Diet Index for US (PHDI-US)	Cacau et al., 2021	16 food components; score ranging from 0 to 150; Adherence expressed proportional 0–10 or 0–5 per food component	Good validity and reliability; Gradations of adherence displayable; Greenhouse gas emissions inversely correlating with higher scores^65^^,^^71^ PHDI-US adapted version of PHDI to display dietary habits of adults in the United States	Based on FFQ from EPIC Oxford; Limited representability of micronutrient intake^65^^,^^71^
Karavasiloglou et al., 2023^a^	EAT-Lancet reference diet index	Knuppel et al., 2019	11 food components; range from 0 to 11; binary scored (0 or 1) for each component, when diet is within recommended range	Ease of calculation, good interpretability	Not all food components of the reference diet are represented^53^; Score can represent a wide variety of dietary scenarios 3/15/25 2:16:00 PM
Liu et al., 2024	EAT-Lancet Diet Score	Knuppel et al., 2019	14 food components; range from 0 to 14; binary scored (0 or 1) for each component, when diet is within recommended range	Ease of calculation, good interpretability	Score can represent a wide variety of dietary scenarios 3/15/25 2:16:00 PM
Mangone et al., 2023^a^	EAT-Lancet Distance Index (EatDI)	Mangone et al., 2023	14 food components; vectors express gaps between recommended and actual for each food component, computation based on mathematical formula expressed as continuous scores	More nuanced measurements of dietary sustainability by penalizing deviations more accurate^27^	Limited comparability to other scores due to its inverse representation of sustainability^27^
Mohammadi et al., 2024^a^	Planetary Health Diet Index (PHDI)	Cacau et al., 2021	16 food components; score ranging from 0 to 150; Adherence expressed proportional 0–10 or 0–5 per food component	Good validity and reliability; Gradations of adherence displayable; Greenhouse gas emissions inversely correlating with higher scores^65^^,^^71^	Based on FFQ from EPIC Oxford; Limited representability of micronutrient intake^65^^,^^71^
Pitt et al., 2024^a^	EAT-Lancet diet Adherence Index (EAI)	Pitt et al., 2024	14 components; range from 0 to 14; Adherence to expressed binary (0 or 1 point) for each food group	Ease of calculation, good interpretability High consistency^54^	Higher scores do not accurately represent higher adherence to the EAT-Lancet diet, higher scores can depict very different diets^54^
Quartiroli et al., 2024^a^	EAT-Lancet Score	Knuppel et al., 2019	12 food components; range from 0 to 12; binary scored (0 or 1) for each component, when diet is within recommended range	Ease of calculation, good interpretability	Not all food components of the reference diet are represented^55^; Score can represent a wide variety of dietary scenarios 3/15/25 2:16:00 PM
Ren et al., 2024	EAT-Lancet Diet Score (ELD)	Stubbendorff et al., 2022	14 food components; Score ranging from 0 to 42; Adherence expressed ordinally (0, 1, 2, or 3 points) per component	High consistency; Good handling and interpretability Greenhouse gas emissions inversely correlating with higher scores^59^^,^^71^	Categorizing food components does not strictly reflect the health-environment balance^59^^,^^71^
Seconda et al., 2020	Sustainable Diet index (SDI)	Seconda et al., 2019	4 subindices: nutritional, environ-mental, economic, sociocultural; each ordinally graduated (1–5 points); total SDI range 4–20	Holistic approach Ease of interpretability^58^^,^^72^^,^^73^	Limited comparability to other scores; Score based on NutriNetSanté (FFQ)^58^^,^^72^^,^^73^
Shan et al., 2025^a^	Planetary Health Diet Index (PHD-I)	Bui et al., 2024	15 food components; Proportionally scored (0–10 or 0–5) per component, total range 0–140	Flexibility and adaptability^40^	Complexity of Interpretation^40^
Stubbendorff et al., 2022^a^	EAT-Lancet Diet Index	Stubbendorff et al., 2022	14 food components; Score ranging from 0 to 42; Adherence expressed ordinally (0, 1, 2, or 3 points) per component	High consistency; Good handling and interpretability Greenhouse gas emissions inversely correlating with higher scores^59^^,^^71^	Categorizing food components does not strictly reflect the health-environment balance
Xiao et al., 2023^a^	EAT-Lancet Diet Score (ELD)	Knuppel et al., 2019	14 food components; range from 0 to 14; binary scored (0 or 1) for each component, when diet is within recommended range	Ease of calculation, good interpretability	Score can represent a wide variety of dietary scenarios 3/15/25 2:16:00 PM
Ye et al., 2023^a^	Planetary Health Diet Score (PHD-S)	Ye et al., 2023	14 food components; each component scored proportionally (0–10 or 0–5) giving a total range of 0–140	Precise graduation of adherence^61^	Restricted representation of diverse global dietary habits^61^
Zhang et al., 2023^a^	Planetary Health Diet Index (PHDI)	Cacau et al., 2021	16 food components; score ranging from 0 to 150; Adherence expressed proportional 0–10 or 0–5 per food component	Good validity and reliability; Gradations of adherence displayable; Greenhouse gas emissions inversely correlating with higher scores^65^^,^^71^	Based on FFQ from EPIC Oxford; Limited representability of micronutrient intake^65^^,^^71^

a Included in the main analysis of the meta-analysis on cancer incidence or mortality.

### Sustainable diets and cancer incidence and cancer mortality

**Fig. 2:**
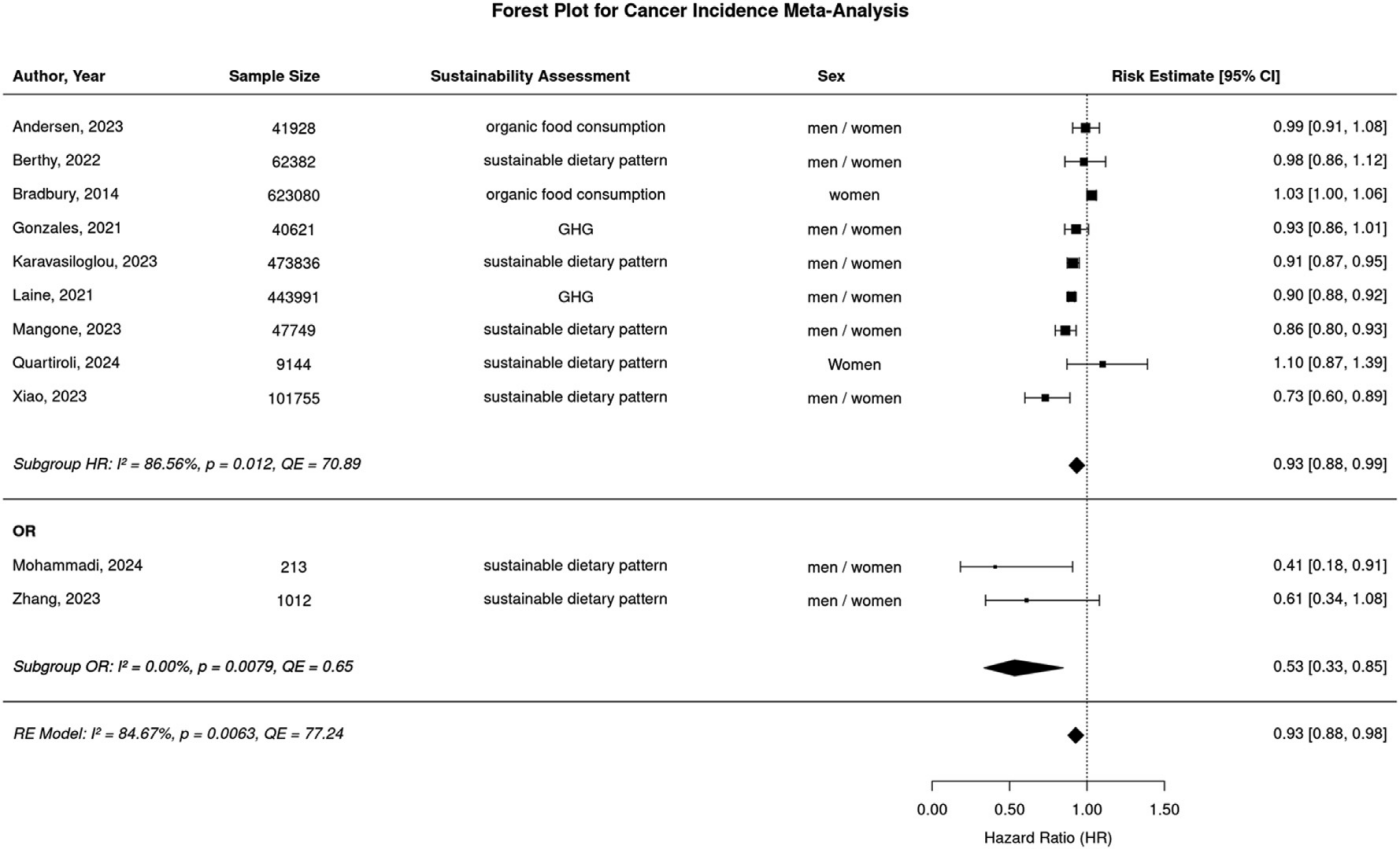
**Forest plot of random effects (RE) meta-analysis for maximally adjusted risk estimates (HR, OR) comparing highest vs. lowest sustainable diets in association to cancer incidence.** Risk estimates and corresponding 95% confidence interval of each study shown as boxes with whiskers. Diamonds indicate summary effect estimate for HR subgroup (RE = 0·911 [CI 95% 0·852–0·973]) and for overall summary effect estimate (RE = 0·911 [CI 95% 0·862–0·962]). I2 and QE represent heterogeneity metrics, p is corresponding p-value).

**Fig. 3:**
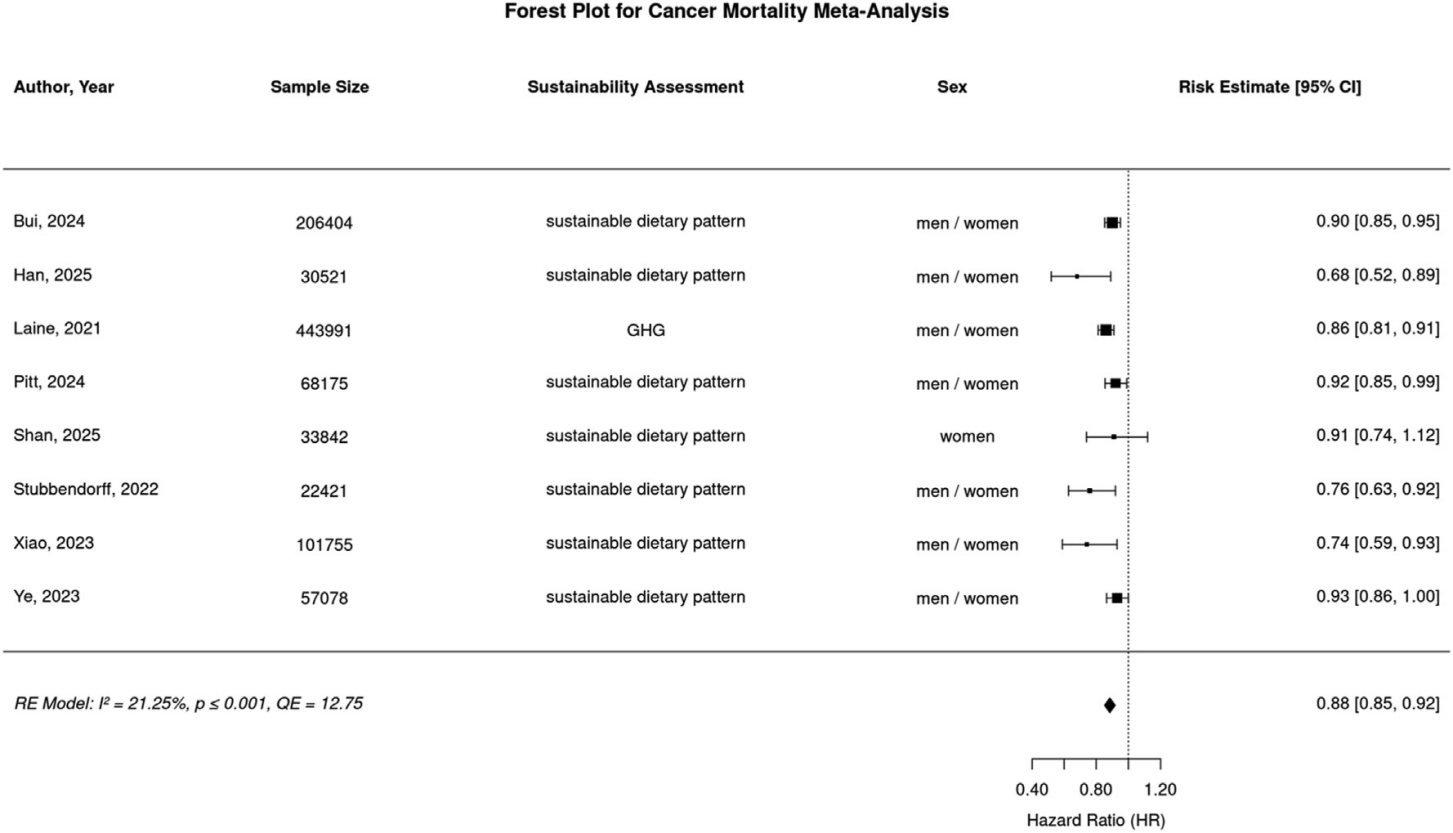
**Forest plot of random effects (RE) meta-analysis for maximally adjusted risk estimates (HR, OR) comparing highest vs. lowest sustainable diets in association to cancer mortality.** Risk estimates and corresponding 95% confidence interval of each study shown as boxes with whiskers. Diamond indicates summary effect estimate for overall risk estimate ((RE = 0·862 [CI 95% 0·805–0·924]). I2 and QE represent heterogeneity metrics, p is corresponding p-value).

For our main analysis, we pooled 19 effect estimates (N = 11 for cancer incidence and N = 8 for cancer mortality) from the 17 included studies. Separate analyses of the two outcomes (cancer incidence and cancer mortality) demonstrated statistically significant inverse associations between high versus low adherence to a sustainable diet and cancer outcomes. The summary effect estimate for cancer incidence indicated an RE of 0·93 [95% CI 0·88–0·98], while cancer mortality revealed an HR of 0·88 [95% CI 0·85–0·92]. Heterogeneity between studies was high (I^2^ = 84·67%) for cancer incidence and moderate (I^2^ = 79·75%) for cancer mortality (Figs. 2 and 3).

### Sensitivity analysis

The overall risk of bias in the included studies was high for four studies due to confounding (Domain 1), exposure measurement (Domain 2), and selection of participants (Domain 3) (Supplemental Figure S1).^28^^,^^29^^,^^50^^,^^64^

Indications of modest potential publication bias for cancer incidence emerged from the asymmetry of the funnel plot (Supplemental Figure S2), supported by Egger's regression test (z = −2·07, p = 0·039). However, Begg's rank correlation test was not significant (p = 1·00; Kendall's tau = −0·02).

For cancer mortality, applied statistics suggests some degree of publication bias (Egger's regression test: z = −2·27, p = 0·023; Begg's rank correlation: p = 0·11; Kendall's tau = −0·50), and indications of modest publication bias was further strengthened visually by funnel plots and trim and fill methods (Supplemental Figure S3). Influence diagnostics for cancer incidence indicated significant effects regarding the study by Bradbury et al.,^50^ but sensitivity analysis did not show significant alterations in the overall summary estimate when leaving this study out (Supplemental Figure S4, Supplemental Table S2). Sensitivity analyses revealed a modest to high influence of the studies by Bui et al.,^40^ Laine et al.,^28^ Pitt et al.,^54^ and Ye et al.^61^ for cancer mortality (Supplemental Tables S3 and S5, Supplemental Figure S5). Despite this, the robustness of the summary effect estimate remained stable, as leave-one-out diagnostics showed no substantial deviations from the summary effect estimate, which ranged from 0·90 [95% CI 0·89–0·92] to 0·94 [95% CI 0·89–0·99] for cancer incidence and from 0·87 [95% CI 0·81–0·92] to 0·89 [95% CI 0·86–0·92] for cancer mortality (Supplemental Tables S2 and S3). When considering all 24 studies within a random effects model meta-analysis for supplemental analysis, the overall effect estimate for cancer incidence was 0·85 [95% CI 0·79; 0·92], and 0·84 [95% CI 0·78; 0·90] for cancer mortality. Sensitivity analysis limited to the inclusion of only one study from the EPIC, NutriNetSanté, PLCO, and UK Biobank cohorts at a time also did not substantially alter the overall risk estimates (Supplemental Tables S4–S7).

The certainty of evidence, influenced by the risk of bias in the included studies, was considered low for both cancer outcomes (Supplemental Tables S8 and S9). To negate the observed summary effect estimates, unmeasured confounders associated with both sustainable diets and cancer would need to have a HR of at least 1·06 (for cancer incidence) and 1·04 (for cancer mortality). For the effect estimates to lose statistical significance, unobserved confounding variables would need effect sizes of 0·06 and 0·04 for cancer incidence and mortality, respectively, to shift the upper confidence limits to include the null value.

Sub-analyses for cancer incidence (Table 3), stratified by study design, sex, adjustment for dietary energy intake, and tumor type, did not reveal substantial changes in the summary effect estimate. However, significant effect modification was observed by stratification based on region (p for difference = 0·0066), study design (p for difference = 0·021), sustainability assessment method (p for difference < 0·0001), and index scoring system (p for difference = 0·041). For cancer mortality, statistically significant effect moderation was observed only for the method of sustainability assessment (p for difference = 0·0086).

**Table 3:** Stratification criteria, risk estimates, summary effect estimates from random-effects meta-regression for each stratum, 95% confidence intervals (CI), subgroup heterogeneity, and between-group differences.

Stratification criteria	Number of included estimates	Effect estimate (highest vs. lowest adherence to a sustainable diet)	95% CI	I^2^	p^difference^ (Cochrane's Q test)
**Cancer incidence**					
Total	11	0·93	0·88–0·98	84·64%	
**Region**	11	0·93	0·88–0·98	84·64%	0·0066^a^
Asia	2	0·96	0·81–1·15	59·02%	
Europe	8	0.92	0·86–0·99	88·59%	
North America	1	0·73	0·60–0·89	0·00%	
**Design**	11	0·93	0·88–0·98	84·64%	0·021^a^
Prospective cohort	9	0·93	0·88–0·99	86·56%	
Case-control	2	0·53	0·33–0·85	0·00%	
**Sex**	10	0·96	0·91–1·01	88·66%	0·90
Men	4	0·96	0·91–1·02	38·12%	
Women	6	0·96	0·89–1·03	90·56%	
**Assessment of sustainability**	14	0·92	0·89–0·96	80·33%	<0.0001^a^
Sustainable dietary pattern	7	0·88	0·80–0·97	67·41%	
Greenhouse gas emissions	3	0·90	0·89–0·92	0·00%	
Land use	2	0·90	0·85–0·94	45·53%	
Organic food consumption	2	1·03	1·00–1·05	0·00%	
**Sustainable diet assessment scoring system**	7	0·88	0·80–0·97	67·41%	0·041^a^
Continuous	2	0·91	0·80–1·03	63·45%	
Binary	3	0·68	0·55–0·85	8·77%	
Proportional	2	0·96	0·81–1·15	59·02%	
**Cancer types**	42	0·92	0·88–0·97	77·99%	0·081
Breast	6	0·94	0·83–1·08	87·40%	
Colorectal	6	0·97	0·92–1·02	0·00%	
Prostate	4	0·96	0·75–1·22	84·93%	
Lung	5	0·87	0·78–0·98	42·06%	
Brain	3	1·03	0·90–1·18	53·92%	
Liver	1	0·79	0·61–1·03	0·00%	
Hematologic (NHL, lymphoma, leukemia)	4	1·04	0·74–1·47	90·11%	
Ovary	2	1·01	0·90–1·14	11·17%	
Esophagus	2	0·63	0·38–1·07	84·10%	
Stomach	3	0·80	0·69–0·94	0·01%	
Pancreas	3	0·95	0·79–1·13	39·50%	
Bladder	3	0·85	0·64–1·15	76·49%	
**Adjustment for dietary energy intake**	11	0·93	0·88–0·98	84·64%	0·89
Adjusted	5	0·93	0·87–0·99	12·35%	
Not adjusted	6	0·92	0·85–0·99	92·28%	
**Cancer mortality**					
Total	8	0·88	0·85–0·92	21·25%	
**Region**	8	0·88	0·85–0·92	21·25%	0·73
Asia	1	0·93	0·86–1·00	0·00%	
Europe	3	0·87	0·81–0·93	48·45%	
North America	4	0·83	0·73–0·95	54·32%	
**Sex**	7	0·89	0·84–0·94	10·18%	0·20
Men	3	0·85	0·79–0·93	0·01%	
Women	4	0·91	0·86–0·93	0·00%	
**Assessment of sustainability**	9	0·87	0·83–0·91	56·79%	0·0086^a^
Sustainable dietary pattern	7	0·90	0·87–0·93	0·04%	
Greenhouse gas emissions	1	0·86	0·81–0·91	0·00%	
Land use	1	0·83	0·80–0·86	0·00%	
**Sustainable diet assessment scoring system**	7	0·90	0·87–0·3	0·04%	0·22
Ordinal	1	0·76	0·63–0·92	0·00%	
Binary	2	0·85	0·69–1·04	68·37%	
Proportional	4	0·90	0·87–0·94	0·19%	
**Adjustment for dietary energy intake**	8	0·88	0·85–0·92	21·25%	0·80
Adjusted	6	0·85	0·78–0·94	64·64%	
Not adjusted	2	0·88	0·84–0·92	22·96%	

a Representing statistical significance.

## Discussion

The present systematic review and meta-analysis aimed to quantify the effects of sustainable diets considering cancer outcomes while identifying modifications in dietary sustainable indicators and comparing dietary scores. A total of 17 studies assessing the association between sustainable diets and cancer outcomes revealed statistically significantly reduced risks of cancer incidence and cancer mortality when comparing the highest dietary sustainability to the lowest rated indicators. Building on a prior systematic review that analyzed nine studies on that issue,^18^ our work contrasts by incorporating quantitative results within a meta-analysis, reporting a moderate reduction in cancer incidence and mortality.

The 2024 *Lancet* Countdown on Health and Climate Change reported an 8% rise in diet-related deaths from 2016 to 2021, while GBD studies estimated that in 2017, 20% of deaths in Western countries were linked to poor nutrition primarily due to inadequate intakes of whole grains, vegetables, fruits, nuts and seeds, and omega-3-fatty acids alongside excessive consumption of sodium, red meat, and dairy.^7^^,^^12^ Studies evaluating the EAT-Lancet diet highlight reduced consumption of animal-based products, especially meat, as critical for minimizing environmental impacts, including land and water GHGs, acidifying and eutrophic pollutants, as even the lowest-impact animal-sourced foods surpass the average environmental footprint of plant-based foods.^6^^,^^74^ The present findings underscore the need for policies promoting healthier, sustainable diets, emphasizing the integration of regional dietary customs, religious traditions, and livestock systems into global recommendations for both environmental and health benefits.^4^^,^^6^^,^^7^^,^^14^^,^^74^

Potential underlying molecular mechanisms of cancer development include the carcinogenic properties of red and processed meats, with the International Agency for Research on Cancer (IARC) linking these foods to colon, stomach, and breast cancers.^4^^,^^75^ Factors contributing to these risks include unfavorable saturated-to-polyunsaturated fat ratios, heme iron, and preservatives such as sodium, nitrates, and nitrites.^4^ In contrast, the Planetary Health Diet promotes nutrient-rich alternatives such as tree nuts, peanuts, legumes (beans, lentils, peas), and soybeans, all of which are rich in unsaturated fats, fiber, vitamins, minerals, antioxidants, and phytosterols, beneficial for reducing cancer risk.^4^ Legumes, in particular soybeans, contain phytochemicals that are structurally mimicries to estrogen and has the potential of reducing the risk of hormone-related cancers like breast cancer.^4^^,^^76^ Additionally, they provide optimal environmental and cost related benefits when considered as milk and meat alternatives in a recent Oxford study.^77^ Whole grains, vegetables, and fruits, which have high density of dietary fiber, offer protection against certain cancers, including colon cancer^78^ by lowering carcinogen absorption, binding cancer-related toxins in the gastrointestinal tract, and reducing plasma levels of hormonal metabolites like estrogens, which are related to breast and endometrial cancers.^55^^,^^78^^,^^79^

Additionally, high-antioxidant foods are found in fruits, especially (blue) berries, legumes, vegetables, and grains and play a critical role in combatting chronic inflammation.^4^^,^^78^^,^^80^ Chronic inflammation is a key factor in carcinogenesis, often exacerbated by high-calorie diets and obesity, which has potential to promote cellular transformation, proliferation, metastasis, and angiogenesis.^80^ Liu et al. and Quartiroli et al. who examined the association between adherence to the EAT-Lancet Diet and cancer also found mediating inflammatory biomarkers to be negatively correlated with higher degrees of adherence, suggesting that this recommended nutrition also has potential anti-inflammatory effects.^55^^,^^67^

Increased consumption of sugar and sweetened beverages disrupts blood sugar and insulin balance, leading to metabolic imbalances, overweight and obesity which are linked to various cancer sites addressed within this study.^4^^,^^78^ Diets high in fiber and nutrient-dense foods do not only help regulate energy intake but also offer protection against obesity, and obesity-linked cancers.^77^^,^^78^ The current evidence linking diet-related causes of cancer with evidence-based nutritional prevention aligns with the principles of sustainable diets. Our findings of decreased cancer incidence and mortality further underscores the importance of promoting sustainable diets as a key strategy for reducing the global burden of cancer.

Subgroup analysis of tumor types detected significant relations between high versus low adherence to sustainable diets and lung and stomach cancer. These findings are linked to evidence suggesting potential inflammatory processes regarding lung cancers and nutrition-derived molecular mechanism of carcinogenesis in terms of gastrointestinal cancers.^4^^,^^60^^,^^67^^,^^75^

Organic food consumption is considered to reflect sustainable diets as it is linked to positive environmental effects, including diminished air and water pollution, lower greenhouse gas emissions, and improved soil quality.^24^, ^25^, ^26^ Within this analysis, we did not find an association between frequency of organic food consumption and cancer and across the studies included for this review, as effects of the assessed consumption were inconsistent.^49^^,^^50^^,^^66^ A recently published meta-analysis on organic food consumption and cancer did not found an association as well, concluding that evidence of a cancer preventive effect of organic nutrition remains uncertain and underlies potential confounding factors that are associated with more health-conscious lifestyle behaviors.^21^ Some organophosphates used in conventional agriculture have been classified as carcinogens,^81^ and a previous meta-analysis found significant associations between organophosphate exposure and NHL risk (OR = 1·22 [95% CI 1·04–1·43]).^82^ In contrast, we found no association, despite the studies reporting NHL as a tumor outcome used organic food consumption as the sustainability assessment but deviated in directions of effect estimates.^50^^,^^66^^,^^83^ Breast cancer has also been linked in current literature to increased pesticide exposure, which is thought to disrupt endocrine function due to toxic residues.^57^^,^^84^, ^85^, ^86^, ^87^ However, associations between pesticide exposure or other forms of adherence to sustainable diets and breast cancer was inconsistent across observational studies,^28^^,^^41^^,^^49^^,^^50^^,^^55^^,^^57^^,^^58^^,^^66^ implying a multifactorial pathogenesis involving additional risk factors.^20^^,^^21^^,^^55^^,^^88^ Overall, the findings concerning cancer incidence are consistent with the health-promoting properties of foods emphasized in sustainable diets, confirming the hypothesis that such diets have cancer-preventive potential. Similarly, considering cancer-related deaths, a meta-analysis restricted to the effects of the EAT-Lancet Diet on mortality reported a 14% reduced cancer-mortality comparing the highest to the lowest adherence to the recommended diet.^19^ These results further highlight the health benefits of recommended sustainable dietary pattern in alignment with this present analysis.

Since the different sustainability metrics used in current literature on sustainable diets rely on distinct measurement scales (e.g., CO_2_ equivalents for greenhouse gas emissions, land use per kg of food, and scoring systems for sustainable dietary patterns), direct comparisons between these aspects are methodologically challenging. Therefore, the meta-analysis adopted an approach that accounts for this heterogeneity while still enabling an overarching interpretation of the results. Sensitivity analyses of cancer incidence and mortality revealed notable variations in pooled risk estimates, depending on the sustainability assessment metrics used.^71^^,^^72^ Similarly, differences in sustainable diet indices significantly modified pooled risk estimates for cancer incidence in this present analysis. For this stratification, we pooled for the referring scoring systems following methods based on Stubbendorff,^59^ Knuppel,^69^ Kesse-Guyot,^68^ Cacau,^70^ and the authors that designed the indices in the studies themselves. Liu et al. conducted a meta-analysis on adherence to the EAT-Lancet Diet and mortality, also facing the challenge of merging different existing scores to assess dietary sustainability. The authors also applied sensitivity analyses due to the variety of scoring systems for cancer outcomes and found similar results as presented in this work.^19^ Most indices focus on environmental impacts and adherence to dietary habits recommended by Willett et al., with limited attention to social sustainability (see Table 2).^4^ Recent evaluations have identified strengths and limitations of existing sustainability scores^71^^,^^72^ (Supplemental Data S3), highlighting the need for standardized sustainability metrics that incorporate environmental footprints and health-promoting food components to enhance generalizability and comparability.^71^^,^^72^

The present systematic review and meta-analysis has some limitations. First, observational studies on this topic are sparse, and those available were conducted in diverse regions with varying dietary cultures. Second, the diversity of dietary sustainability assessment methods limited direct comparisons, necessitating cautious interpretation of results. These methodological differences contribute to heterogeneity in effect estimates and highlight the need for standardized approaches to sustainability assessment in nutritional epidemiology. Merging sustainability metrics is challenging, as some focus solely on environmental impact (GHG, land use) while others combine both planetary and human health (dietary scores). Several studies reporting associations between environmental sustainability and cancer outcomes also conducted correlation analyses linking environmental metrics to adherence to the EAT-Lancet diet^28^ or reported both environmental and dietary sustainability scores.^27^ For example, Gonzales et al.^29^ linked lower GHG to reduced meat and higher vegetable intake, aligning with the principles of the Planetary Health Diet.^4^ Third, risk of bias in the included studies was moderate to high, primarily due to confounding factors and reliance on self-reported food frequency data, which are susceptible to imprecise exposure measurements, recall errors, and social desirability bias. As a result, the overall GRADE assessment rated the certainty of evidence as “low”. Fourth, dietary behaviors from (large-scale) observational studies rather reflect snapshots of specific time points of dietary assessments and may not have captured dietary changes over time. Fifth, a limitation of our analysis is the heterogeneity in how energy intake was handled across the studies which may impact the comparability and accuracy of the findings as some studies adjusted for energy intake while others did. Sixth, our analysis included only observational studies representing populations from high-income countries in the Global North, based on volunteers who may be more health-conscious, limiting the generalizability of our findings to the broader global population.

While social dimensions are integral to sustainable diets per FAO guidelines, higher adherence to sustainable dietary behaviors often correlates with higher socioeconomic status and healthier lifestyle factors, which is a general limitation of sustainable diets that should be minded.^49^^,^^50^^,^^57^^,^^66^^,^^73^

Despite these limitations, the present work provides notable strengths. To the best of our knowledge, this systematic review and meta-analysis is the first to separately quantify associations between broader aspects of sustainable diets and cancer incidence and cancer mortality. The present work includes a wide range of specific parameters to assess dietary sustainability and reaches beyond examinations of existing meta-analyses. The health-conscious profile of study participants suggests that the associations observed could be even greater in the general population. In addition, we analyzed biochemical mechanisms linking dietary food components to carcinogenesis either promoting or inhibiting cancer development, aligned with recommendations for sustainable diets.

Our findings align with existing research on the health benefits of sustainable diets, which are associated with reduced risk and mortality of NCDs, such as cardiovascular disease,^89^ overweight and obesity,^17^^,^^47^ and type 2 diabetes.^90^ Sustainable diets play an important rule within SDGs, encompassing systemic sustainability efforts such as waste reduction, advanced farming practices, ecosystem preservation, and social equity.^1^^,^^4^^,^^91^

Conclusively, the findings of this present analysis showed reduced incidence and mortality of cancer when compared highest to lowest adherence to sustainable diets. From a planetary health perspective, the findings of this study emphasize the need for policies that promote sustainable food environments–including in the health sector–to support sustainable diets for the dual purposes of health promotion and cancer prevention while maintaining environmental balance.^1^^,^^4^^,^^6^^,^^14^ Such initiatives are vital to meeting the needs of a growing global population in a sustainable and equitable manner.

## Contributors

CJ and MK created the concept of this work and performed the statistical analyses and interpretation of the data. MM contributed to the data extraction process by verifying the underlying study data and supported the risk of bias assessment. TK contributed to evaluation of the overall evidence of this study. MK drafted the initial manuscript with supportive input from CJ. MK and CJ had full access to the underlying data and verified them. All authors critically reviewed and revised the manuscript for intellectual content and ensured its accuracy and completeness. They had full access to the study's data and collectively took final responsibility for deciding to submit the manuscript for publication.

## Data sharing statement

The data underlying this meta-analysis were obtained from publicly available published sources. No individual participant data were collected. Additional analyses and summary datasets generated supporting this study's findings are available from the corresponding author upon reasonable request.

## Declaration of interests

The authors declare no competing interests of relevance for the contents of this work.
