# Supplementary material for: Sustainable diets and cancer: a systematic review and meta-analysis
Source: eClinicalMedicine. 2025 Apr 29;83:103215. doi: 10.1016/j.eclinm.2025.103215 (PMC12235399; doi:10.1016/j.eclinm.2025.103215)
Supplement: Supplemental Materials [file mmc1.docx]

**Supplementary Appendix**

Marina Kasper^1^, Mirna al Masri^2^, Tilman Kühn^3,4,5,6^, Sabine Rohrmann^7^, Katharina Wirnitzer^8,9,10,11^, Michael Leitzmann^1^, Carmen Jochem^1,2^

^1^ Department of Epidemiology and Preventive Medicine, University of Regensburg, Regensburg, Germany

^2^ Chair of Planetary & Public Health, University of Bayreuth, Bayreuth, Germany

^3^ Center for Public Health, Medical University of Vienna, Vienna, Austria

^4^ Department of Nutritional Sciences, University of Vienna, Vienna, Austria

^5^ Heidelberg Institute of Global Health (HIGH), Medical Faculty and University Hospital, Heidelberg University, Heidelberg, Germany

^6^ Institute of Global Food Security (IGFS), Queen's University Belfast, Belfast, UK

^7^ Division of Chronic Disease Epidemiology, Epidemiology, Biostatistics and Prevention Institute, University of Zurich, Zurich, Switzerland

^8^ Department of Pediatric Oncology and Hematology, Otto-Heubner Centre for Paediatric and Adolescent Medicine (OHC), Charité – Universitätsmedizin Berlin, Augustenburger Platz 1, 13353 Berlin, Germany;

^9^ Charité Competence Center for Traditional and Integrative Medicine (CCCTIM), Charité – Universitätsmedizin Berlin, Berlin, Germany

^10^ Department of Sport Science, Leopold-Franzens University of Innsbruck, Fürstenweg 185, 6020 Innsbruck, Austria;

^11^ Department of Secondary Education, University College of Teacher Education Tyrol, Pastorstraße 7, 6010 Innsbruck, Austria;

[Supplemental Data 1: Search Terms applied to PubMed, ISI Web of Science, Cochrane Library and EMBASE 3](#_Toc192940169)

[Supplemental Data 2: Study excluded at full-text screening stage with reasons for exclusion 3](#_Toc192940170)

[Supplemental Data 3: Evaluation of common sustainable diet indices and scores used for included studies 3](#_Toc192940171)

[Supplemental Figure 1: Results from assessment of Risk of bias evaluated with the ROBINS-E tool 6](#_Toc192940172)

[Supplemental Figure 2: Funnel plot for assessment of risk of publication bias for cancer incidence analysis (trim and fill method) 7](#_Toc192940173)

[Supplemental Figure 3: Funnel plot for assessment of risk of publication bias for cancer mortality analysis (trim and fill method) 7](#_Toc192940174)

[Supplemental Figure 4: Influence diagnostics of studies assessing sustainable diets and cancer incidence 8](#_Toc192940175)

[Supplemental Figure 5: Influence diagnostics of studies assessing sustainable diets and cancer mortality 8](#_Toc192940176)

[Supplementary Table 1: PRISMA Checklist 9](#_Toc192940177)

[Supplemental Table 2: Leave-one-out analysis and influence diagnostics of studies assessing sustainable diets and cancer incidence 12](#_Toc192940178)

[Supplemental Table 3: Leave-one-out analysis and influence diagnostics of studies assessing sustainable diets and cancer mortality 12](#_Toc192940179)

[Supplemental Table 4 Sensitivity analysis for the studies based on the NutriNet-Santé cohort; only one effect estimate was included in the supplementary model of overall estimate of cancer incidence (summary risk estimate with n=17 studies RE=0·85 [95% CI 0·79; 0·92], I](#_Toc192940180)^[2](#_Toc192940180)^[=94·56%, p <0·0001 12](#_Toc192940180)

[Supplemental Table 5: Sensitivity analysis for the studies based on the EPIC cohort; only one effect estimate was included in the supplementary model of overall estimate of cancer mortality (summary risk estimate with n=10 studies RE=0·84 [95% CI 0·78; 0·90], I](#_Toc192940181)^[2](#_Toc192940181)^[=75·48%, p <0·0001 12](#_Toc192940181)

[Supplemental Table 6: Sensitivity analysis for the studies based on the EPIC cohort; only one effect estimate was included in the supplementary model of overall estimate of cancer incidence (summary risk estimate with n=17 studies RE=0·85 [95% CI 0·79; 0·92], I](#_Toc192940182)^[2](#_Toc192940182)^[=94·56%, p <0·0001 13](#_Toc192940182)

[Supplemental Table 7: Sensitivity analysis for the studies based on the PLCO cohort; only one effect estimate was included in the supplementary model of overall estimate of cancer incidence (summary risk estimate with n=17 studies RE=0·85 [95% CI 0·79; 0·92], I](#_Toc192940183)^[2](#_Toc192940183)^[=94·56%, p <0·0001 13](#_Toc192940183)

[Supplemental Table 8: GRADE Assessment of studies examining sustainable diets and cancer incidence 14](#_Toc192940184)

[Supplemental Table 9: GRADE Assessment of studies examining sustainable diets and cancer mortality 14](#_Toc192940185)

**Supplemental Data 1: Search Terms applied to PubMed, ISI Web of Science, Cochrane Library and EMBASE**

| **PubMed 615 results (February 28, 2025)** |
| --- |
| **("cancer"[All Fields] OR "neoplasms"[MeSH Terms]) AND ("planetary health"[All Fields] OR "sustainable diet"[All Fields] OR "food biodiversity"[All Fields] OR "sustainable lifestyle"[All Fields] OR "organic food consumption"[All Fields] OR "organic food"[All Fields] OR "sustainable food"[All Fields] OR "greenhouse gas"[All Fields] OR "greenhouse gasses"[All Fields] OR "GHG"[All Fields] OR "environmental footprint"[All Fields])** |
| **Web of Science 164 results (February 28, 2025)** |
| **((((((((((((ALL=(sustainable)) OR ALL=(sustainable diet)) OR ALL=(PHDI)) OR ALL=(organic food)) OR ALL=(sustainable food)) OR ALL=(greenhouse gasses)) OR ALL=(GHG))OR ALL=(planetary health)) OR ALL=(food biodiversity) AND ALL=(diet)) AND ALL=(neoplasm)) AND ALL=(cancer)))** |
| **EMBASE 625 results (February 28, 2025)** |
| **('cancer' OR 'neoplasms'/exp) AND ('planetary health' OR 'sustainable diet' OR 'food biodiversity' OR 'sustainable lifestyle' OR 'organic food consumption' OR 'organic food' OR 'sustainable food' OR 'greenhouse gas' OR 'greenhouse gases' OR 'GHG' OR 'environmental footprint')** |
| **Cochrane Library (February 28, 2025)** |
| ("cancer" OR "neoplasm*") AND ("planetary health" OR "sustainable diet" OR "food biodiversity" OR "sustainable lifestyle" OR "organic food consumption" OR "organic food" OR "sustainable food" OR "greenhouse gas" OR "greenhouse gases" OR "GHG" OR "environmental footprint") |

**Supplemental Data 2: Study excluded at full-text screening stage with reasons for exclusion**

| Study, excluded after full-text screening | Reason for exclusion |
| --- | --- |
| Chen H, Wang X, Ji JS, Huang L, Qi Y, Wu Y, He P, Li Y, Bodirsky BL, Müller C, Willett WC, Yuan C. Plant-based and planetary-health diets, environmental burden, and risk of mortality: a prospective cohort study of middle-aged and older adults in China. Lancet Planet Health. 2024 Aug;8(8):e545-e553. doi: 10.1016/S2542-5196(24)00143-8. PMID: 39122323.  Zhan J, Bui L, Hodge RA, Zimmer M, Pham T, Rose D, Willits-Smith A, Willett WC. Planetary Health Diet Index Trends and Associations with Dietary Greenhouse Gas Emissions, Disease Biomarkers, Obesity, and Mortality in the United States (2005-2018). Am J Clin Nutr. 2025 Jan 9:S0002-9165(25)00007-3. doi: 10.1016/j.ajcnut.2025.01.007. Epub ahead of print. PMID: 39793707. | No data shown on cancer mortality, but overall mortality  No data shown on cancer mortality, but overall mortality |

**Supplemental Data 3: Evaluation of common sustainable diet indices and scores used for included studies**

To measure sustainability of dietary behaviors the authors considered different indices that reflect adherence to food components recommended by the EAT-Lancet commission by either binary, ordinal^1–5^, proportional or continuous^6,7^ scoring systems^8^ according to scores that are based on Stubbendorff^3^, Knuppel^9^, Kesse-Guyot^10^, Cacau^11^ and the authors that designed the indices in the studies themselves. Some studies did not assess all the food components that are recorded for the scores, resulting in adapted ranges of the scores^12,13^. There is no existing evidence that one certain score is better than the other which might result from their heterogenous computation systems but they showed distinct differences in terms of their comparability, interpretability and handling.^14^ Recent systematic evaluations identified strengths and limitations of different current sustainability scores^14,15^ four of which^3,10,11,16^ were used for quantifying sustainability of diets in the included studies used for this review. Based on the nature of the score, binary and ordinal scales offer good consistency and ease of use and interpretation. While continuous scores allow for more precise classification of adherence, they are difficult to interpret and may even offset adherence to recommended foods with increased consumption of food components that should be more limited in intake. Binary scoring can indicate vastly different diets and must not accurately reflect dietary behaviors that adhere to the EAT-Lancet recommendations.^14^ Proportional scores that have wider ranges as they evaluate adherence to recommended consumption of a certain food component have the tendency to be more precisely than binary or ordinally scaled scores that reflect a smaller range to capture sustainability of an individual’s diet.^4,17^ Most of the scores used in studies of this review aimed to assess adherence to the reference diet based on food components that are categorized as either emphasized or limited, recommended by Willet et al.^8^ but lack in evaluating social aspects of a sustainable dietary pattern except for one.^15^ The Sustainable Diet Index (SDI) based on Seconda et al. holds a special position because it does not only reflect diet-related environmental effects but also considers social sustainability by taking economic and sociocultural aspects into account. Findings from that study stated that a higher SDI is correlated to higher socioeconomic status and more health favorable lifestyle and dietary patterns. Hence, the authors highlighted a potential limitation of sustainable diets in terms of their affordability, at least based on the French population their study is based on.^16^

1. Zhang W, He Y, Wang C, Chen F, Jiang B, Li W. Adherence to Healthy Dietary Patterns and Glioma: A Matched Case-Control Study. Nutrients. 23. November 2023;15(23):4886.

2. Seconda L, Baudry J, Allès B, Touvier M, Hercberg S, Pointereau P, u. a. Prospective associations between sustainable dietary pattern assessed with the Sustainable Diet Index (SDI) and risk of cancer and cardiovascular diseases in the French NutriNet-Santé cohort. Eur J Epidemiol. Mai 2020;35(5):471–81.

3. Stubbendorff A, Sonestedt E, Ramne S, Drake I, Hallström E, Ericson U. Development of an EAT-Lancet index and its relation to mortality in a Swedish population. Am J Clin Nutr. 4. März 2022;115(3):705–16.

4. Ye YX, Geng TT, Zhou YF, He P, Zhang JJ, Liu G, u. a. Adherence to a Planetary Health Diet, Environmental Impacts, and Mortality in Chinese Adults. JAMA Netw Open. 2. Oktober 2023;6(10):e2339468.

5. Bui LP, Pham TT, Wang F, Chai B, Sun Q, Hu FB, u. a. Planetary Health Diet Index and risk of total and cause-specific mortality in three prospective cohorts. Am J Clin Nutr. Juli 2024;120(1):80–91.

6. Mangone L, Sacerdote C, Laine J, Masala G, Bendinelli B, Panico S, u. a. Food, Health, and Mitigation of Climate change in Italy. Epidemiol Prev. 2023;47(3):32–8.

7. Berthy F, Brunin J, Allès B, Fezeu LK, Touvier M, Hercberg S, u. a. Association between adherence to the EAT-Lancet diet and risk of cancer and cardiovascular outcomes in the prospective NutriNet-Santé cohort. Am J Clin Nutr. 6. Oktober 2022;116(4):980–91.

8. Willett W, Rockström J, Loken B, Springmann M, Lang T, Vermeulen S, u. a. Food in the Anthropocene: the EAT–Lancet Commission on healthy diets from sustainable food systems. The Lancet. 2. Februar 2019;393(10170):447–92.

9. Knuppel A, Papier K, Key TJ, Travis RC. EAT-Lancet score and major health outcomes: the EPIC-Oxford study. The Lancet. 20. Juli 2019;394(10194):213–4.

10. Kesse-Guyot E, Rebouillat P, Brunin J, Langevin B, Allès B, Touvier M, u. a. Environmental and nutritional analysis of the EAT-Lancet diet at the individual level: insights from the NutriNet-Santé study. J Clean Prod. 10. Mai 2021;296:126555.

11. Cacau LT, De Carli E, de Carvalho AM, Lotufo PA, Moreno LA, Bensenor IM, u. a. Development and Validation of an Index Based on EAT-Lancet Recommendations: The Planetary Health Diet Index. Nutrients. Mai 2021;13(5):1698.

12. Quartiroli M, Roncallo C, Pala V, Simeon V, Ricceri F, Venturelli E, u. a. Adherence to Diet Quality Indices and Breast Cancer Risk in the Italian ORDET Cohort. Nutrients. Januar 2024;16(8):1187.

13. Karavasiloglou N, Thompson AS, Pestoni G, Knuppel A, Papier K, Cassidy A, u. a. Adherence to the EAT-Lancet reference diet is associated with a reduced risk of incident cancer and all-cause mortality in UK adults. One Earth Camb Mass. 15. Dezember 2023;6(12):1726–34.

14. Stubbendorff A, Stern D, Ericson U, Sonestedt E, Hallström E, Borné Y, u. a. A systematic evaluation of seven different scores representing the EAT–Lancet reference diet and mortality, stroke, and greenhouse gas emissions in three cohorts. Lancet Planet Health. 1. Juni 2024;8(6):e391–401.

15. Neta RS de O, Lima SCVC, Nascimento LL do, Souza CVS de, Lyra C de O, Marchioni DML, u. a. Indices for measurement of sustainable diets: A scoping review. PLOS ONE. 20. Dezember 2023;18(12):e0296026.

16. Seconda L, Baudry J, Pointereau P, Lacour C, Langevin B, Hercberg S, u. a. Development and validation of an individual sustainable diet index in the NutriNet-Santé study cohort. Br J Nutr. Mai 2019;121(10):1166–77.

17. Shan Y, Bertrand KA, Petrick JL, Sheehy S, Palmer JR. Planetary Health Diet Index in relation to mortality in a prospective cohort study of United States Black females. Am J Clin Nutr. 1. März 2025;121(3):589–96.

**Supplemental Figure 1: Results from assessment of Risk of bias evaluated with the ROBINS-E tool**


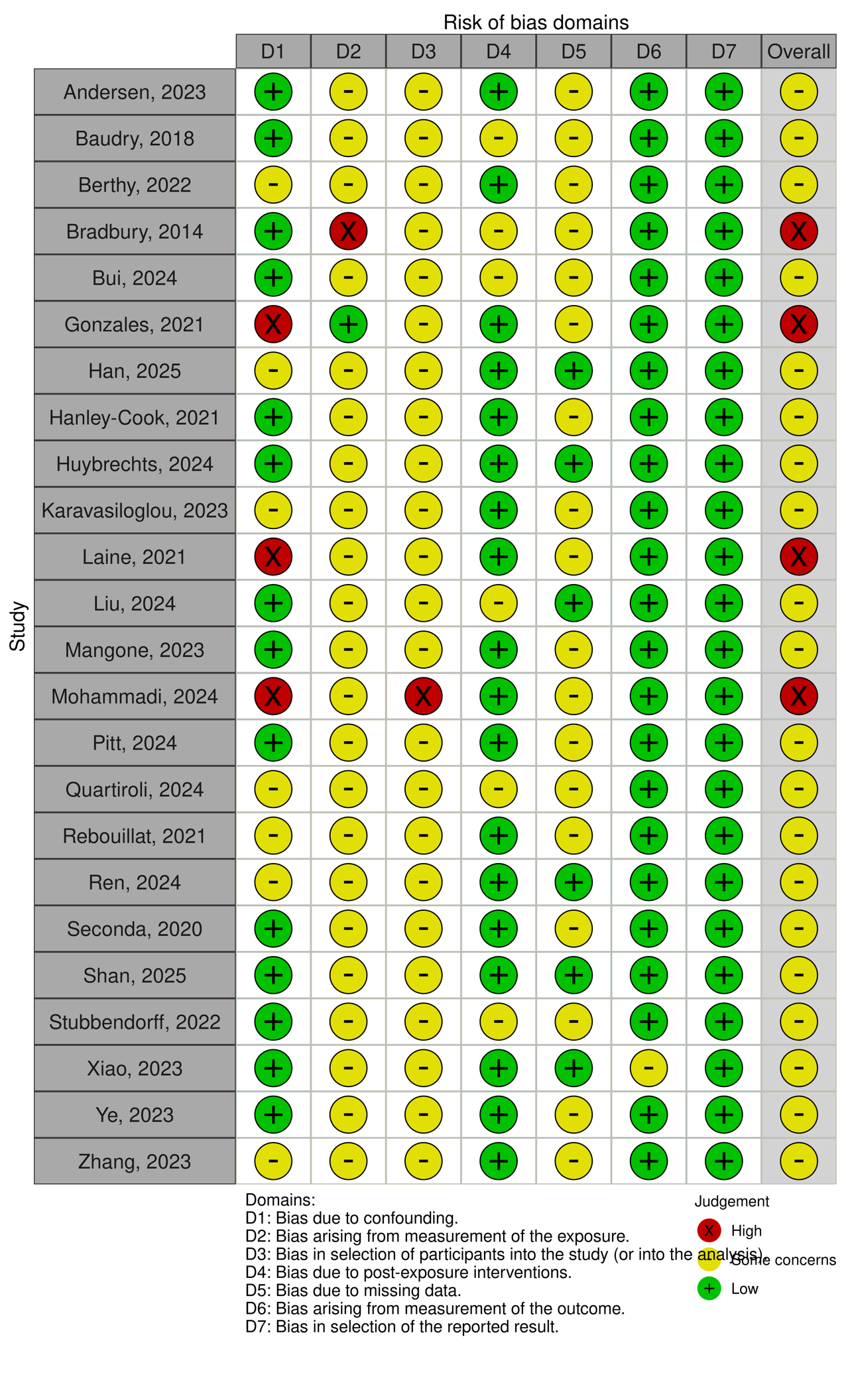


**Supplemental Figure 2: Funnel plot for assessment of risk of publication bias for cancer incidence analysis (trim and fill method)**

**
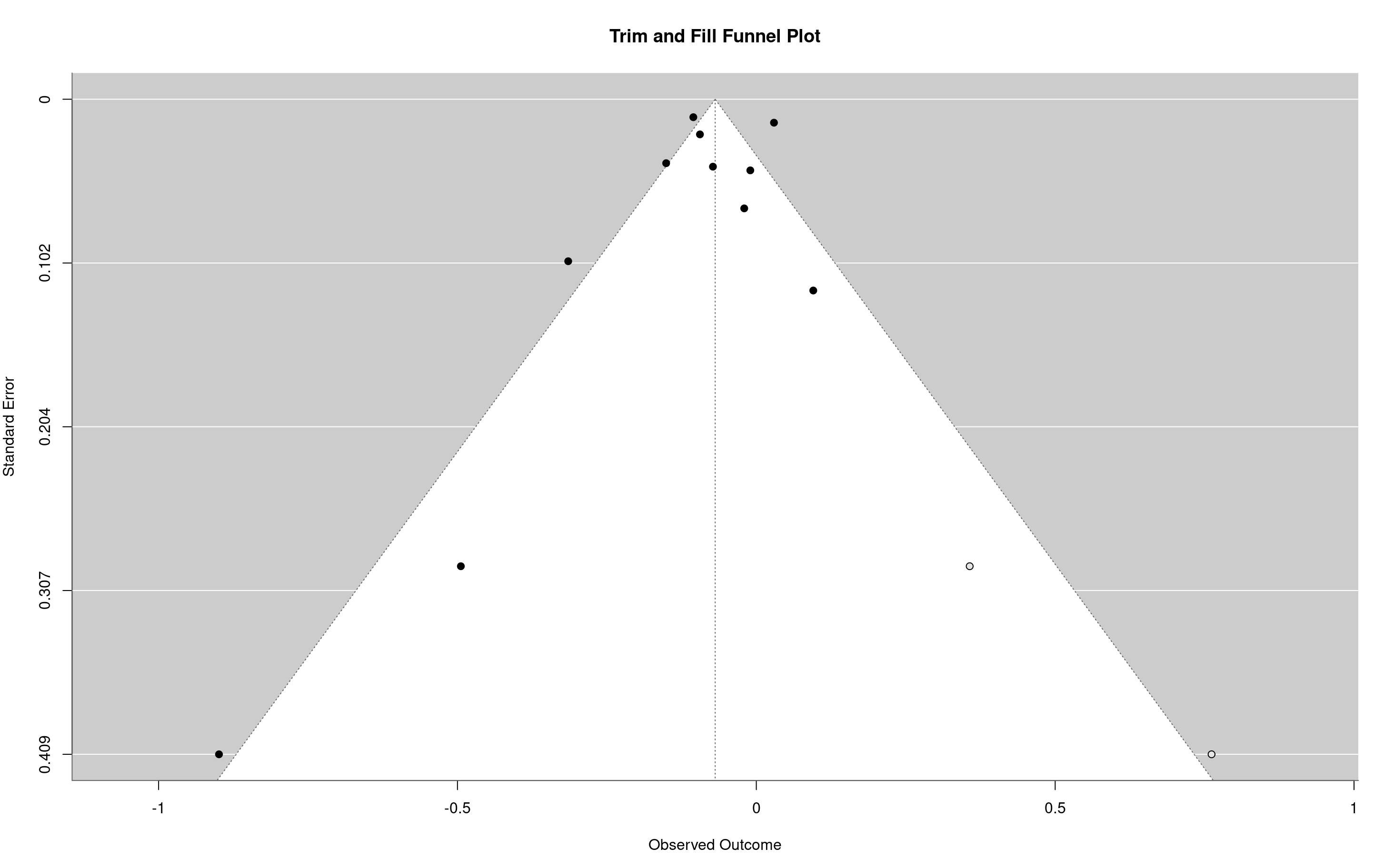
**

**Supplemental Figure 3: Funnel plot for assessment of risk of publication bias for cancer mortality analysis (trim and fill method)**


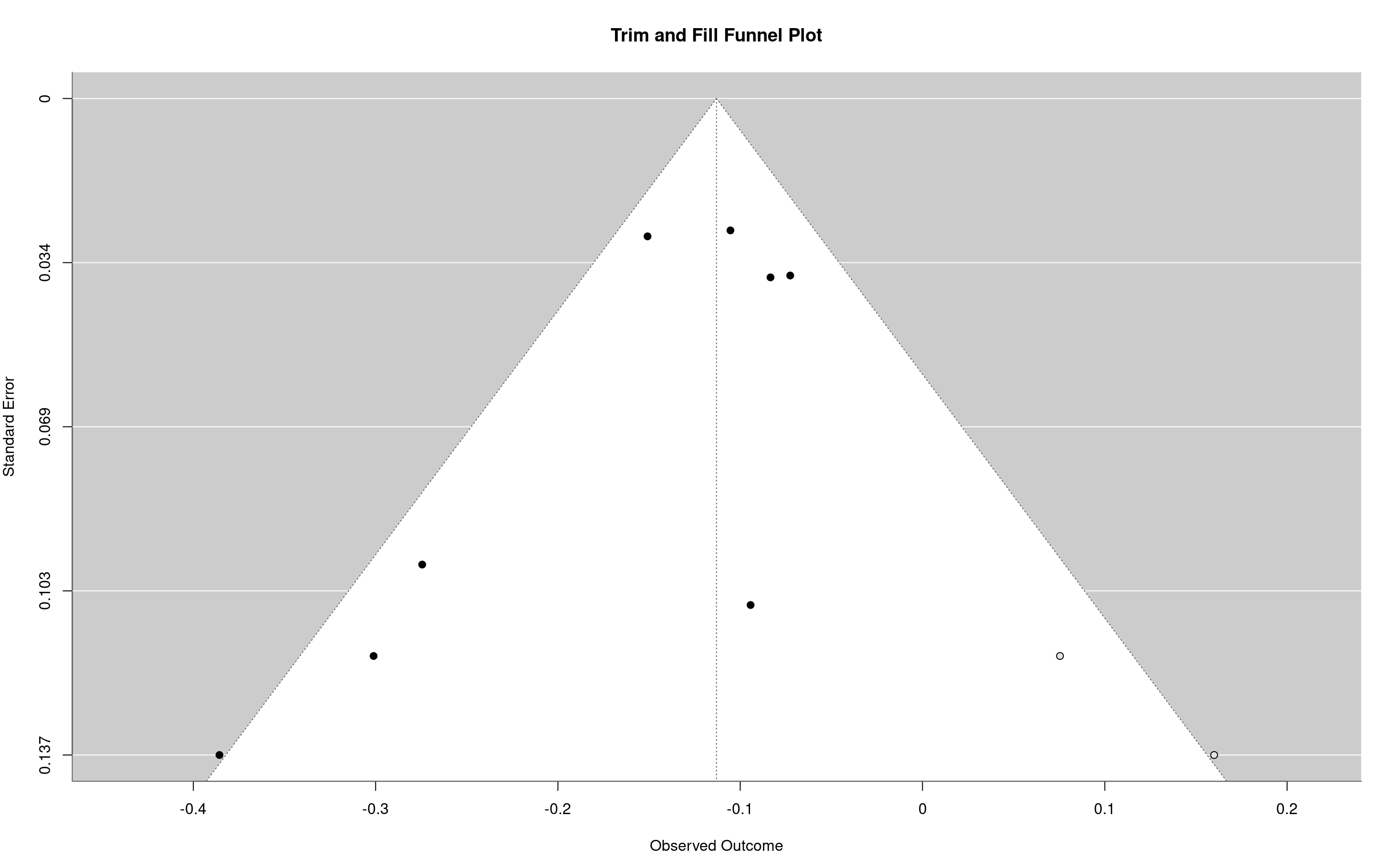


**Supplemental Figure 4: Influence diagnostics of studies assessing sustainable diets and cancer incidence**


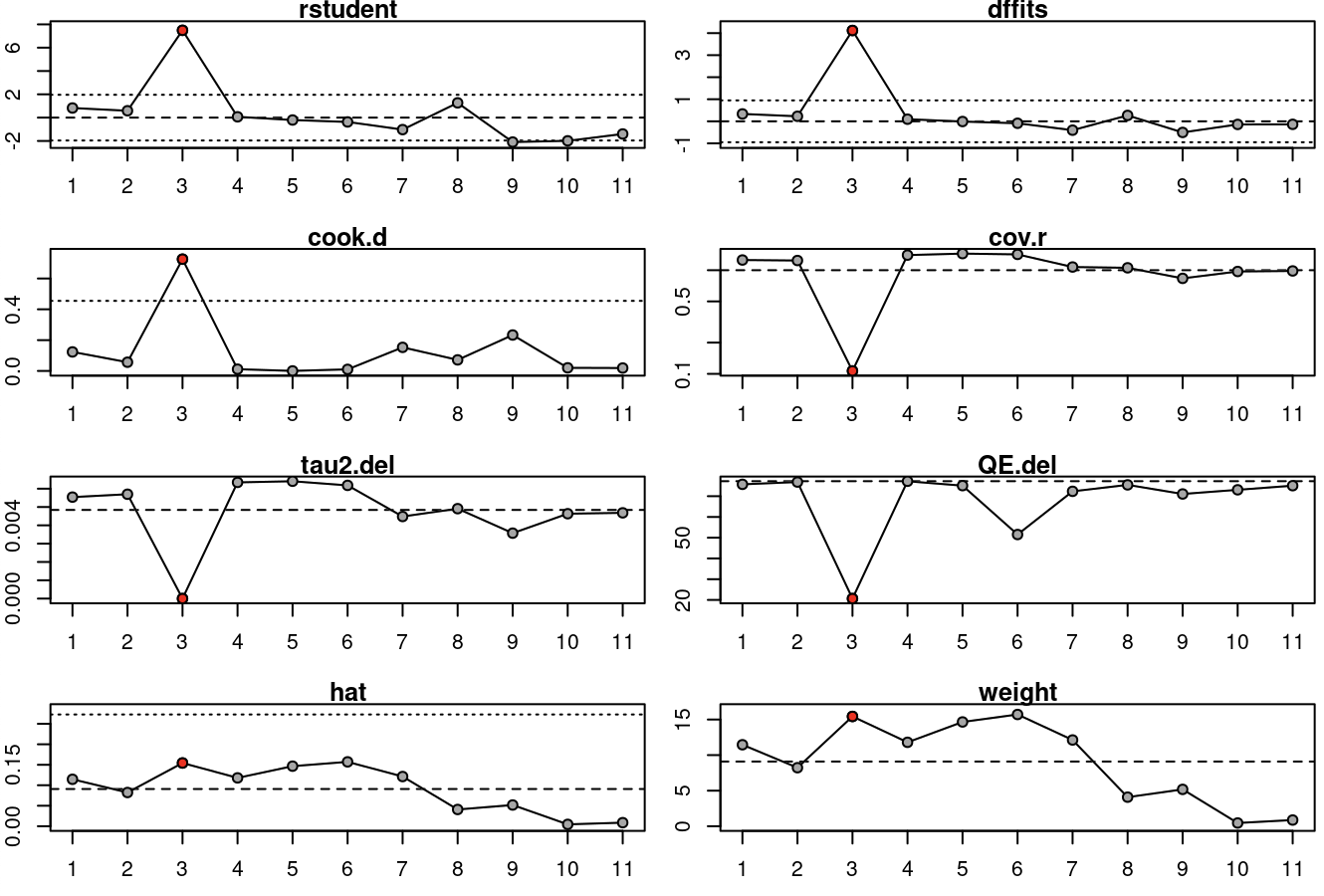


1=Andersen, 2023, 2= Berthy, 2022, 3= Bradbury, 2014,

4 = Gonzales, 2021, 5= Karavasiloglou, 2023, 6= Laine, 2021, 7= Mangone, 2023, 8= Mohammadi, 2024, 9= Quartiroli, 2024, 10= Xiao, 2023, 11= Zhang, 2023

**Supplemental Figure 5: Influence diagnostics of studies assessing sustainable diets and cancer mortality**


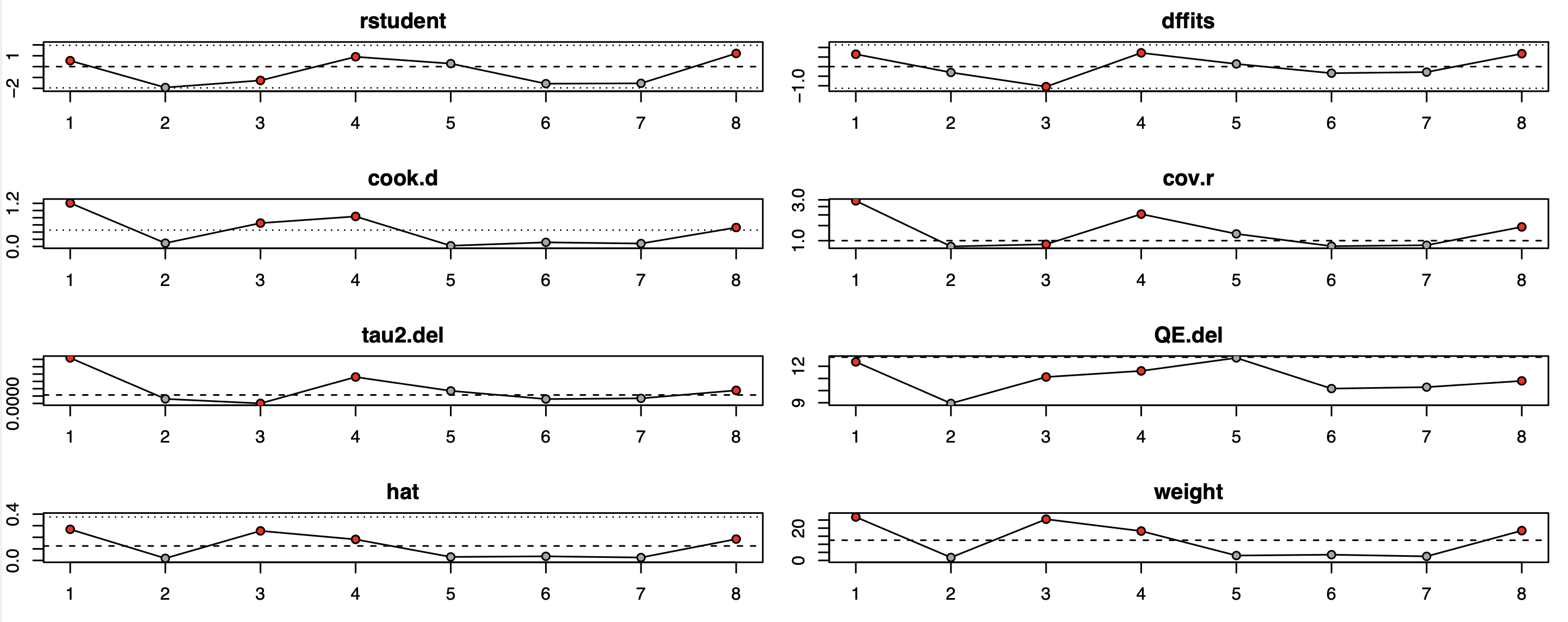


1= Bui, 2024, 2= Han, 2025, 3=Laine, 2021, 4= Pitt, 2024, 5= Shan, 2025, 6= Stubbendorff, 2022, 7= Xiao, 2023, 8= Ye, 2023

**Supplementary Table 1: PRISMA Checklist**

| **Section and Topic** | **Item #** | **Checklist item** | **Location where item is reported (p)** |
| --- | --- | --- | --- |
| **TITLE** | | |  |
| Title | 1 | Identify the report as a systematic review. | 1 |
| **ABSTRACT** | | |  |
| Abstract | 2 | See the PRISMA 2020 for Abstracts checklist. | 2 |
| **INTRODUCTION** | | |  |
| Rationale | 3 | Describe the rationale for the review in the context of existing knowledge. | 5 |
| Objectives | 4 | Provide an explicit statement of the objective(s) or question(s) the review addresses. | 6 |
| **METHODS** | | |  |
| Eligibility criteria | 5 | Specify the inclusion and exclusion criteria for the review and how studies were grouped for the syntheses. | 7 |
| Information sources | 6 | Specify all databases, registers, websites, organisations, reference lists and other sources searched or consulted to identify studies. Specify the date when each source was last searched or consulted. | 7-8 |
| Search strategy | 7 | Present the full search strategies for all databases, registers and websites, including any filters and limits used. | 7-8 Supplemental Data 1 |
| Selection process | 8 | Specify the methods used to decide whether a study met the inclusion criteria of the review, including how many reviewers screened each record and each report retrieved, whether they worked independently, and if applicable, details of automation tools used in the process. | 8-9, Figure 1, Supplemental Data 2 |
| Data collection process | 9 | Specify the methods used to collect data from reports, including how many reviewers collected data from each report, whether they worked independently, any processes for obtaining or confirming data from study investigators, and if applicable, details of automation tools used in the process. | 8 |
| Data items | 10a | List and define all outcomes for which data were sought. Specify whether all results that were compatible with each outcome domain in each study were sought (e.g. for all measures, time points, analyses), and if not, the methods used to decide which results to collect. | 8-9 |
|  | 10b | List and define all other variables for which data were sought (e.g. participant and intervention characteristics, funding sources). Describe any assumptions made about any missing or unclear information. | 8-9 |
| Study risk of bias assessment | 11 | Specify the methods used to assess risk of bias in the included studies, including details of the tool(s) used, how many reviewers assessed each study and whether they worked independently, and if applicable, details of automation tools used in the process. | 9-10, Supplemental Figure 1, 2, and 3 |
| Effect measures | 12 | Specify for each outcome the effect measure(s) (e.g. risk ratio, mean difference) used in the synthesis or presentation of results. | 8-9 |
| Synthesis methods | 13a | Describe the processes used to decide which studies were eligible for each synthesis (e.g. tabulating the study intervention characteristics and comparing against the planned groups for each synthesis (item #5)). | 8-9, Figure 1, Supplemental Data 2 |
|  | 13b | Describe any methods required to prepare the data for presentation or synthesis, such as handling of missing summary statistics, or data conversions. | 8-9 |
|  | 13c | Describe any methods used to tabulate or visually display results of individual studies and syntheses. | 8-9, Figure 2 and 3 |
|  | 13d | Describe any methods used to synthesize results and provide a rationale for the choice(s). If meta-analysis was performed, describe the model(s), method(s) to identify the presence and extent of statistical heterogeneity, and software package(s) used. | 8-9 |
|  | 13e | Describe any methods used to explore possible causes of heterogeneity among study results (e.g. subgroup analysis, meta-regression). | 9-10 |
|  | 13f | Describe any sensitivity analyses conducted to assess robustness of the synthesized results. | 9-10 |
| Reporting bias assessment | 14 | Describe any methods used to assess risk of bias due to missing results in a synthesis (arising from reporting biases). | 5 |
| Certainty assessment | 15 | Describe any methods used to assess certainty (or confidence) in the body of evidence for an outcome. | 5, 9, Supplementary Figure 1, Supplemental Table 8 and 9 |
| **RESULTS** | | |  |
| Study selection | 16a | Describe the results of the search and selection process, from the number of records identified in the search to the number of studies included in the review, ideally using a flow diagram. | 10, Figure 1 |
|  | 16b | Cite studies that might appear to meet the inclusion criteria, but which were excluded, and explain why they were excluded. | Supplemental Data 2 |
| Study characteristics | 17 | Cite each included study and present its characteristics. | 11-12,Table 1 |
| Risk of bias in studies | 18 | Present assessments of risk of bias for each included study. | Supplemental Figure 1, 2 and 3 |
| Results of individual studies | 19 | For all outcomes, present, for each study: (a) summary statistics for each group (where appropriate) and (b) an effect estimate and its precision (e.g. confidence/credible interval), ideally using structured tables or plots. | 18-22, Table 1, Figure 2 and 3 |
| Results of syntheses | 20a | For each synthesis, briefly summarise the characteristics and risk of bias among contributing studies. | 18-19, Figure 2 and 3 |
|  | 20b | Present results of all statistical syntheses conducted. If meta-analysis was done, present for each the summary estimate and its precision (e.g. confidence/credible interval) and measures of statistical heterogeneity. If comparing groups, describe the direction of the effect. | 18-22, Table 3, Supplemental Appendix |
|  | 20c | Present results of all investigations of possible causes of heterogeneity among study results. | 18-19, Supplemental Appendix |
|  | 20d | Present results of all sensitivity analyses conducted to assess the robustness of the synthesized results. | 18-22, Supplemental Appendix |
| Reporting biases | 21 | Present assessments of risk of bias due to missing results (arising from reporting biases) for each synthesis assessed. | Supplemental Appendix |
| Certainty of evidence | 22 | Present assessments of certainty (or confidence) in the body of evidence for each outcome assessed. | 18-22, Supplemental Appendix |
| **DISCUSSION** | | |  |
| Discussion | 23a | Provide a general interpretation of the results in the context of other evidence. | 24 |
|  | 23b | Discuss any limitations of the evidence included in the review. | 28-29 |
|  | 23c | Discuss any limitations of the review processes used. | 28-29 |
|  | 23d | Discuss implications of the results for practice, policy, and future research. | 24,25-27, 29-30 |
| **OTHER INFORMATION** | | |  |
| Registration and protocol | 24a | Provide registration information for the review, including register name and registration number, or state that the review was not registered. | 7 |
|  | 24b | Indicate where the review protocol can be accessed, or state that a protocol was not prepared. | 7 |
|  | 24c | Describe and explain any amendments to information provided at registration or in the protocol. | 7 |
| Support | 25 | Describe sources of financial or non-financial support for the review, and the role of the funders or sponsors in the review. | 29 |
| Competing interests | 26 | Declare any competing interests of review authors. | 29 |
| Availability of data, code and other materials | 27 | Report which of the following are publicly available and where they can be found: template data collection forms; data extracted from included studies; data used for all analyses; analytic code; any other materials used in the review. | Supplemental Appendix |

*From:*  Page MJ, McKenzie JE, Bossuyt PM, Boutron I, Hoffmann TC, Mulrow CD, et al. The PRISMA 2020 statement: an updated guideline for reporting systematic reviews. BMJ 2021;372:n71. doi: 10.1136/bmj.n71. This work is licensed under CC BY 4.0. To view a copy of this license, visit <https://creativecommons.org/licenses/by/4.0/>

**Supplemental Table 2: Leave-one-out analysis and influence diagnostics of studies assessing sustainable diets and cancer incidence**

|  | Leave-one-out-analysis | | Influence diagnostics | | | |
| --- | --- | --- | --- | --- | --- | --- |
| Study | Estimate | CI 95% | R-student | DFFITS | Cook‘s Distance | Weight |
| Andersen, 2023 | 0·9175 | [0·8630–0·9755] | 0·8520 | 0·3345 | 0·123 | 11·45% |
| Berthy, 2022 | 0·9204 | [0·8660–0·9783] | 0·5896 | 0·2277 | 0·0566 | 8·23% |
| Bradbury, 2014 | 0·9048 | [0·8888–0·9211] | 7·4895* | 4·1182* | 0·7254* | 15·43%* |
| Gonzales, 2021 | 0·9238 | [0·8659–0·9856] | 0·0694 | 0·0959 | 0·0113 | 11·80% |
| Karavasiloglou, 2023 | 0·9269 | [0·8678–0·9899] | -0·2052 | -0·0101 | 0·0001 | 14·66% |
| Laine, 2021 | 0·9292 | [0·8705–0·9918] | -0·3706 | -0·0893 | 0·0101 | 15·71% |
| Mangone, 2023 | 0·9368 | [0·8851–0·9914] | -1·0281 | -0·4025 | 0·1530 | 12·13% |
| Mohammadi, 2024 | 0·9197 | [0·8694–0·9729] | 1·2665 | 0·2672 | 0·0716 | 4·09% |
| Quartiroli, 2024 | 0·9392 | [0·8934–0·9873] | -2·0960 | -0·5045 | 0·2331 | 5·18% |
| Xiao, 2023 | 0·9303 | [0·8814–0·9818] | -1·9900 | -0·1418 | 0·0201 | 0·45% |
| Zhang, 2023 | 0·9301 | [0·8810–0·9820] | -1·4031 | -0·1372 | 0·0188 | 0·87% |

*statistical significance

**Supplemental Table 3: Leave-one-out analysis and influence diagnostics of studies assessing sustainable diets and cancer mortality**

|  | Leave-one-out-analysis | | Influence diagnostics | | | |
| --- | --- | --- | --- | --- | --- | --- |
| Study | Estimate | CI 95% | R-student | DFFITS | Cook‘s Distance | Weight |
| Bui, 2024 | 0·8662 | [0·8130–0·9230] | 0·55* | 0·65* | 1·21* | 26·87%* |
| Han, 2025 | 0·8896 | [0·8596–0·9207] | -1·93 | -0·31 | 0·09 | 1·85% |
| Laine, 2021 | 0·8981 | [0·8669–0·930] | -1·27* | -1·05* | 0·65* | 25·53%* |
| Pitt, 2024 | 0·8694 | [0·8243–0·9167] | 0·90* | 0·72* | 0·84* | 18·18%* |
| Shan, 2025 | 0·8821 | [0·8470–0·9188] | 0·28 | 0·14 | 0·02 | 3·05% |
| Stubbendorff, 2022 | 0·8902 | [0·8600–0·9214] | -1·57 | -0·34 | 0·11 | 3·56% |
| Xiao, 2023 | 0·8893 | [0·8587–0·9209] | -1·54 | -0·29 | 0·08 | 2·53% |
| Ye, 2023 | 0·8724 | [0·8344–0·9123] | 1·21* | 0·68* | 0·53%* | 18·45%* |

*statistical significance

**Supplemental Table 4 Sensitivity analysis for the studies based on the NutriNet-Santé cohort; only one effect estimate was included in the supplementary model of overall estimate of cancer incidence (summary risk estimate with n=17 studies RE=0·85 [95% CI 0·79; 0·92], I^2^=94·56%, p <0·0001**

| Included study | Overall Risk Estimate | p | I^2^ |
| --- | --- | --- | --- |
| Baudry et al, 2018 | 0·85 [95% CI 0·78; 0·93] | 0·00030 | 94·65% |
| Berthy et al, 2022 | 0·87 [95% CI 0·80; 0·94] | 0·0010 | 94·12% |
| Rebouillat et al, 2021 | 0·87 [95% CI 0·80; 0·94] | 0·00050 | 93·85% |
| Seconda et al, 2020 | 0·83 [95% CI 0·74; 0·92] | 0·00050 | 96·39% |

**Supplemental Table 5: Sensitivity analysis for the studies based on the EPIC cohort; only one effect estimate was included in the supplementary model of overall estimate of cancer mortality (summary risk estimate with n=10 studies RE=0·84 [95% CI 0·78; 0·90], I^2^=75·48%, p <0·0001**

| Included study | Overall Risk Estimate | p | I^2^ |
| --- | --- | --- | --- |
| Hanley-Cook et al, 2021 | 0·82 [95% CI 0·76; 0·89] | <0·0001 | 74·38% |
| Laine et al, 2021 | 0·87 [95% CI 0·83; 0·91] | <0·0001 | 41·75% |

**Supplemental Table 6: Sensitivity analysis for the studies based on the EPIC cohort; only one effect estimate was included in the supplementary model of overall estimate of cancer incidence (summary risk estimate with n=17 studies RE=0·85 [95% CI 0·79; 0·92], I^2^=94·56%, p <0·0001**

| Included study | Overall Risk Estimate | p | I^2^ |
| --- | --- | --- | --- |
| Huybrechts et al, 2024 | 0·84 [95% CI 0·76; 0·92] | 0·00040 | 91·41% |
| Laine et al, 2021 | 0·86 [95% CI 0·80; 0·93] | <0·0001 | 93·54% |

**Supplemental Table 7: Sensitivity analysis for the studies based on the PLCO cohort; only one effect estimate was included in the supplementary model of overall estimate of cancer incidence (summary risk estimate with n=17 studies RE=0·85 [95% CI 0·79; 0·92], I^2^=94·56%, p <0·0001**

| Included study | Overall Risk Estimate | p | I^2^ |
| --- | --- | --- | --- |
| Xiao, 2023 | 0·87 [95% CI 0·81; 0·94] | 0·00020 | 92·62% |
| Ren, 2024 | 0·84 [95% CI 0·77; 0·91] | <0·0001 | 94·64% |

**Supplemental Table 8: GRADE Assessment of studies examining sustainable diets and cancer incidence**

|  | **Downgrading** | | | | | **Upgrading** | **Overall assessment** |
| --- | --- | --- | --- | --- | --- | --- | --- |
| Factor | Risk of Bias | Inconsitency | Indirectness | Imprecision | Publication bias | Effect size;  Dose-response gradient;  Effect of potential residual confounding |  |
| Assessment | Downgraded by two levels | Down-  graded by one level | Not downgraded | Not downgraded | Not downgraded | Upgraded by one | Low  ⨁◯◯◯ |
| Comment | High risk of bias in four studies | High heteroge-neity, not all effect estimates show same direction | Population based studies | Large studies, rather tight confidence intervals | Statistical tests and Funnel plots showed modest indication for publication bias, with only a slight trend of stronger associations from smaller studies | Studies showed strong correlation between adherence level to more sustainable diets and cancer outcome |  |

**Supplemental Table 9: GRADE Assessment of studies examining sustainable diets and cancer mortality**

|  | **Downgrading** | | | | | **Upgrading** | **Overall assessment** |
| --- | --- | --- | --- | --- | --- | --- | --- |
| Factor | Risk of Bias | Inconsitency | Indirectness | Imprecision | Publication bias | Effect size;  Dose-response gradient;  Effect of potential residual confounding |  |
| Assessment | Downgraded by two levels | Not downgraded | Not downgraded | Not downgraded | Not downgraded | Upgraded by one | Low  ⨁◯◯◯ |
| Comment | High risk of bias in one study | Despite high heterogeneity, all effect estimates show same direction | Population based studies | Large studies, rather tight confidence intervals | Statistical tests and funnel plots showed modest indication for publication bias, with only a slight trend of stronger associations from smaller studies | Studies showed strong correlation between adherence level to more sustainable diets and cancer outcome |  |
